# Heavy metals in soils of Mayabeque, Cuba: multifaceted and hardly discernable contributions from pedogenic and anthropogenic sources

**DOI:** 10.1007/s10661-022-10097-6

**Published:** 2022-05-20

**Authors:** Dayana Sosa, Isabel Hilber, Diane Buerge-Weirich, Roberto Faure, Arturo Escobar, Thomas D. Bucheli

**Affiliations:** 1 Centro Nacional de Sanidad Agropecuaria (CENSA), Apartado 10, CP32700, San José de Las Lajas, Mayabeque, Cuba; 2 Environmental Analytics, Agroscope, Reckenholzstrasse 191, CH-8046 Zurich, Switzerland

**Keywords:** Cuba, Heavy metals, Monitoring, Soils, Anthropogenic influence, Pedogenic sources

## Abstract

Cuba is a country with considerable potential for economic growth, and special efforts are made to increase the agricultural output. As food production depends on the quality of soils, heavy metal concentrations were measured in 39 soils in the province of Mayabeque, Cuba, and interpreted in light of anthropogenic activities and pedogenic conditions (soil type and properties). With median concentrations of 1.8 Cd, 60.3 Cr, 48.1 Cu, 36.2 Ni, 16.7 Pb, 55.0 Zn, and 0.1 mg/kg Hg, soils of Mayabeque were mostly below Cuban quality reference values (QRV) representing benchmarks of quality standards but no official threshold values. Only Cd concentrations were in many cases above the QRV of 0.6 mg/kg and some Cu concentrations above the one of 83 mg/kg. While Cd, Cr, and Ni concentrations were rather pedogenically driven, Cu, Pb, Zn, and Hg contents were rather anthropogenically influenced. When evaluated statistically, Cd and Cr showed most times a significant influence of both sources. In contrast, Ni and Zn could not be significantly related with the origins investigated in this study. Hence, the allocation of heavy metal concentrations to pedogenic or anthropogenic contamination or pollution sources is tentative and needs further investigations. Nevertheless, the present data adds information on soil heavy metal concentrations in the Caribbean region, serves as reference before further industrial development, and sets the ground for adaptation of the QRV for Cd and possibly future national environmental standards.

**Supplementary Information:**

The online version contains supplementary material available at 10.1007/s10661-022-10097-6.

## Introduction

Anthropogenic sources, such as industry or agriculture, as well as natural processes like pedogenesis, are responsible for the input of heavy metals to soils (Li et al., 2013). Trace metals, such as cobalt (Co), copper (Cu), iron (Fe), manganese (Mn), molybdenum (Mo), nickel (Ni), vanadium (V), and zinc (Zn), are needed in a certain concentration for the development of organisms, but are toxic at an overdose. However, metals like lead (Pb), cadmium (Cd), and mercury (Hg) are considered as major threat, as they have no beneficial effect and are harmful to plants and animals even at low concentrations (Chibuike & Obiora, 2014). Metals are not degradable and the residence time in soil is long compared to air or water. Depending on the conditions, they can accumulate in the soil or can be (re)mobilised. In this case, metals either are transported by soil water to the groundwater or are taken up by plants and, thus, enter the food chain.

As it is very difficult to remediate soils polluted with heavy metals, it is important to protect them and to survey their metal concentrations (Chapman, 2007; Xia et al., 2004). Many countries try to achieve this by regulations and by defining threshold values for metal concentrations in soils (Agroscope, 2020; Ballesta et al., 2010; Desaules, 2012). This has not been done in Cuba yet, but quality reference values (QRV) exist as benchmarks of soil quality (Rodríguez et al., 2015). As agriculture and good soil quality are very important for Cuban economy (Machado, 2012), soil pollution (Chapman, 2007) is considered by the Ministry of Science, Technology and Environment (CITMA) as one of the main environmental problems (CITMA, 2015). Food and crop productions are essential for domestic use and export like coffee and sugar. Some researchers have determined concentrations of heavy metals in different soil types of Cuba with little human activity (Amaral et al., 2013; Pérez et al., 2012; Rodríguez et al., 2015). However, the spatial distribution of heavy metals, considering different anthropogenic influences, or different soil types or land uses, has never been investigated before.

**Fig. 1:**
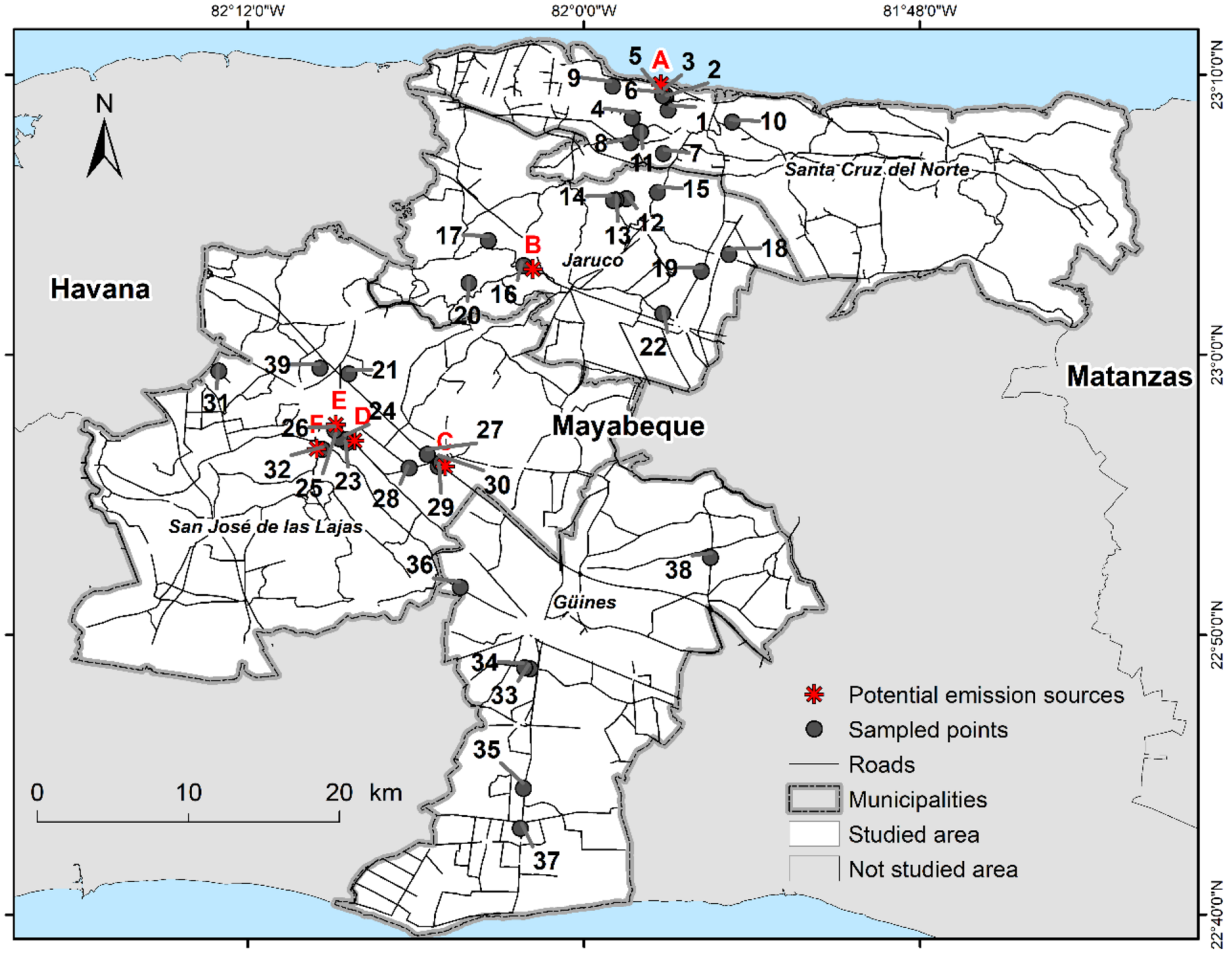
Soil sampling sites in the four municipalities of Santa Cruz del Norte (SC), San José de las Lajas (SJ), Jaruco (JA), and Güines (GU) in Mayabeque. The sites are marked with black dots and numbered (1–39). Presumed major emission sources are marked with a red star and a red capital letter specifying the emission source types: thermoelectric power plant (**A**), zeolite production (**B**), asphalt factory (**C**), cable industry (**D**), rubber manufactory (**E**), waste incineration (**F**). Reprinted and adapted with permission from Sosa et al. Springer

Mayabeque is a province southeast of Havana (Fig. 1), in which industry and agriculture co-exist at short distances. As such, it is a model province for the whole country and ideally suited to carry out a monitoring programme for pollutants in Cuba, as recently illustrated for persistent organic pollutants (Sosa et al., 2017). About 383,400 inhabitants live in this region that has an area of about 3,744 km^2^. Agricultural land includes 2,568 km^2^ (68.6% of the total area) of which 1,346 km^2^ is cultivated (ONEI, 2018). The north, Santa Cruz del Norte (SC), is mostly industrialised, with fossil fuel (9,351,171 t in 2017) and gas (681,791 thousand m^3^ in 2017) production (ONEI, 2017). The cultivation of vegetables is also quite important with 7,404 t in 2017 (ONEI, 2017). In Jaruco (JA), the main activity is textile production (552,000 units in 2015) and the municipality could be described as rural (ONEI, 2015). San José de las Lajas (SJ) hosts several secondary sector industries that produce, e.g., wires and cables (30,092 km in 2017) or ceramic tiles (180,250 m^2^ in 2017) and there was vegetable production of 27,761 t in 2017 (ONEI, 2017). The southern municipality of Güines (GU) is mainly agriculturally influenced and the vegetable production is higher (82,079 t in 2017; ONEI, 2017) in comparison to SC and SJ.

The aims of this study were to determine heavy metal concentrations in soils and to attribute them to major pollutant sources in the four municipalities (SC, SJ, JA and GU) of the province of Mayabeque. In detail, and for the first time in Cuba, we discuss the influence of different soil types, properties and land uses on the concentration of heavy metals and try to relate concentration gradients to different sources of pollution. For that, statistical tools were used such as analysis of variance (ANOVA) and pairwise comparisons, linear regressions and descriptive evaluations such as principal component analysis (PCA), a predictive model based on iron content (Hamon et al., 2004; Rodríguez et al., 2015), and literature studies of Cuban pedologists (Hernández-Jiménez, 2021; Hernández-Jiménez et al., 2015, 2019), all this with the purpose to distinguish anthropogenic influences from pedogenic or natural background concentrations.

## Material and methods

### Area under investigation

Samples for this study were taken in four municipalities in the province of Mayabeque, Cuba. Briefly, the area and number of inhabitants for these municipalities are 379 km^2^ and 32,756 for SC, 276 km^2^ and 24,602 for JA, 594 km^2^ and 78,227 for SJ, and 435 km^2^ and 66,446 for GU (ONEI, 2017). Mayabeque has five weather locations which recorded a humid tropical climate with a minimum and maximum median temperature of 19.8 °C and 30.0 °C and a median annual rainfall of 1,447 mm in 2018 (ONEI, 2018). The soil types of the sampling sites were determined by Hernández-Jiménez et al. (2015) and Lopez-Kramer et al. (2012) (electronic supplementary information ESI, Table S1 in chapter S1) and belong to the six reference soil groups (RSG, Table S2) (leptosol, cambisol, regosol, nitisol, vertisol, and gleysol) classified by the World Reference Base (WRB) for soil resources 2014 (IUSS Working Group WRB, 2015). A description of the RSG, their management and use and qualifying properties according to the WRB (IUSS Working Group WRB, 2015) are provided in Table S2. Please note that the land use in Table S3 refers to the actual cultivation of the sites at the sampling, whereas Table S2 column “management and use” informs about the use recommended due to soil types, which did not necessarily coincide.

### Soil sampling and sample pre-treatment

Soil samples that were collected between January and April 2014 during the dry season in 39 different sites in the above-mentioned municipalities (Fig. 1), as described in Sosa et al. (2017), were used for analysis. The main site selection criteria were the municipalities, soil type, land use (crop cultivation, forest, and pasture; Table S3), and human activity reflected by the potential pollution sources (Table S3, Fig. 1). In contrast to these anthropogenically influenced sites, negative control sites were sampled (sites 8, 9, 21, 31 and 39; Table S3) where potential polluting sources were assumed to be too remote to be of influence. Further information is given in Table S3.

Soil sampling was performed according to the method used in the Swiss National Soil Monitoring Network (NABO), described in detail in Hämmann and Desaules (2003). On an area of 10 m × 10 m, 100 soil subsamples were taken, i.e. one for each m^2^, with a steel gouge auger (3 cm inside diameter, 0–20 cm depth, including the humus layer). Twenty-five of these subsamples, evenly distributed over the 100 m^2^, were mixed together to get four bulk samples out of the 100 m^2^. They were put in a polyethylene bag and transported in a cool box to the laboratory where they were transferred into paper bags and dried at 40 °C. Afterwards, they were sieved through a 2-mm mesh to isolate the fine earth fraction and stored in the dark in polyethylene bottles at 20–25 °C. An aliquot of 100 g was taken out of each sample after extended shaking and shipped to Agroscope, Switzerland, for analysis.

### Extraction and analysis of Cd, Cr, Cu, Ni, Pb, Zn, and Hg in soils

The soil samples were extracted according the Swiss Reference Method HNO_3_-EX (Agroscope, 2020). Nitric acid (100 mL, 2 M) was added to 10 g of fine earth. This mixture was put for exactly 2 h in a cooking water bath and filtrated afterwards (folded filters 1291, diameter 185 mm of Munktell, Bärenstein, Germany). The extracts were measured for Cd, Cr, Cu, Ni, Pb, and Zn on an inductively coupled plasma-optical emission spectrometer (ICP-OES). The instrument used was the Arcos from Spectro (Kleve, Germany) equipped with a V-groove nebulizer, a Scott spray chamber and radial Argon plasma. The samples were measured undiluted. The calibration range for these elements was between 0.1 and 30 mg/L with a linear behaviour for all the elements. The wavelengths for Cd were 228.80 nm, Cr 267.16 nm, Cu 324.75 nm, Ni 231.60 nm, Pb 220.35 nm and Zn 206.20 nm due to their optical interferences, precision and sensitivity. Physical interferences in the plasma were corrected by using yttrium as internal standard; the correction was checked by monitoring the argon line.

Mercury was measured by cold vapor-atomic fluorescence spectroscopy (AFS) on a Mercur instrument from Analytik Jena AG (Jena, Germany). Before measurement, the extracts were diluted 10 times and organic mercury was oxidised by BrCl to Hg(II). Afterwards, the samples were mixed online with SnCl_2_ to reduce Hg(II) to gaseous Hg(0), which was separated from the aqueous phase in a liquid–gas separator, dried in a membrane and measured directly by fluorescence at 253.7 nm (Agroscope, 2020). The calibration curve covered the range from 0.04 to 4 µg Hg/L and was linear. For Hg analysis, very pure acids and chemicals had to be used to avoid contamination (Plasma Pure Acids from SCP (Baie d’Urfé, Canada)). All the other chemicals were from VWR (Radnor, PA, USA).

### pH, organic carbon (C_org_) and texture measurements

Soil pH, C_org_ and texture were measured according their corresponding Swiss Reference methods that are accessible via the weblink (Agroscope, 2020). Briefly, for pH, 15 g of the fine earth was equilibrated overnight (12 to 18 h) with 50 mL water and measured automatically with a Robotic titrosampler from Metrohm, Herisau, Switzerland, equipped with a special pH flat membrane electrode for suspensions (6.0256.100 from Metrohm). The calibration was done by pH buffers with pH 4.0 and 7.0.

For the measurement of C_org_, 0.3 g of soil is mixed in surplus with dichromate. For oxidation, the suspension is put for 7 min in a sand bath, heated to 210 °C. Afterwards, the remaining amount of dichromate is titrated with an Fe(II) solution, and thus, the C_org_ present in the soil can be calculated over the oxidation equation, assuming that the oxidation state of C_org_ is 0.

To define the texture, the amount of clay and silt was measured with a pipetting method. Clay is defined in Switzerland as particles below 2 µm and 50 μm. Briefly, the suspensions (10 g of soil, in which C_org_ was removed before due to oxidation by H_2_O_2_, in 500 mL water) were agitated, and after defined times, exactly 25 mL of suspension was sampled at a defined depth. The dry amount of this subsample was determined, and out of this, the amount of clay and silt could be calculated using Stokes’ law. Sand, i.e. particles > 50 µC_org_ by 1.725), clay, silt and sand represents 100% of the soil.

### Quality control

The method detection (MDL) and quantification limits (MQL) of all heavy metals were calculated from blank values (extraction with HNO_3_ without soil). The former was defined as mean of the blank values plus three times the standard deviation and the latter corresponded to the mean plus ten times the standard deviation. The MDL varied between 0.04 and 1.9 mg/kg and the MQL between 0.1 and 2.7 mg/kg. Detailed values are listed in Table S4.

This extraction method was originally developed in the Agroscope laboratory for Swiss soils. As the Fe contents in the Cuban soils were much higher than those in Swiss soils and could lead to precipitation of heavy metals, such as Cu, we checked its robustness for such conditions by spiking the extracts of Cuban soils with different concentrations of Cu (6 mg/mL and 19 mg/mL corresponding to an expected average concentration range in samples and Cu to be a representative metal because it can easily precipitate in the presence of Fe). The recovery of Cu was 105% ± 3% for all spiked samples indicating no precipitation of heavy metals by soil Fe.

Internal quality control was based on the soil sample number 863 from the Wageningen Evaluation Programmes for Analytical Laboratories (WEPAL, Wageningen, Netherlands). It was measured at least once in each series. Recoveries of the certified values of these soils of all elements analysed ranged between 90 and 108%. In each series, blank samples, i.e. acid samples without soil, but treated the same way as soil samples, were measured. Their values were always below the MQL (Table S4). External quality control of the Agroscope laboratory was performed by regular successful participation in the ISE (International Soil Exchange) programme of WEPAL.

### Data presentation, statistical evaluations and modelling

**Fig. 2:**
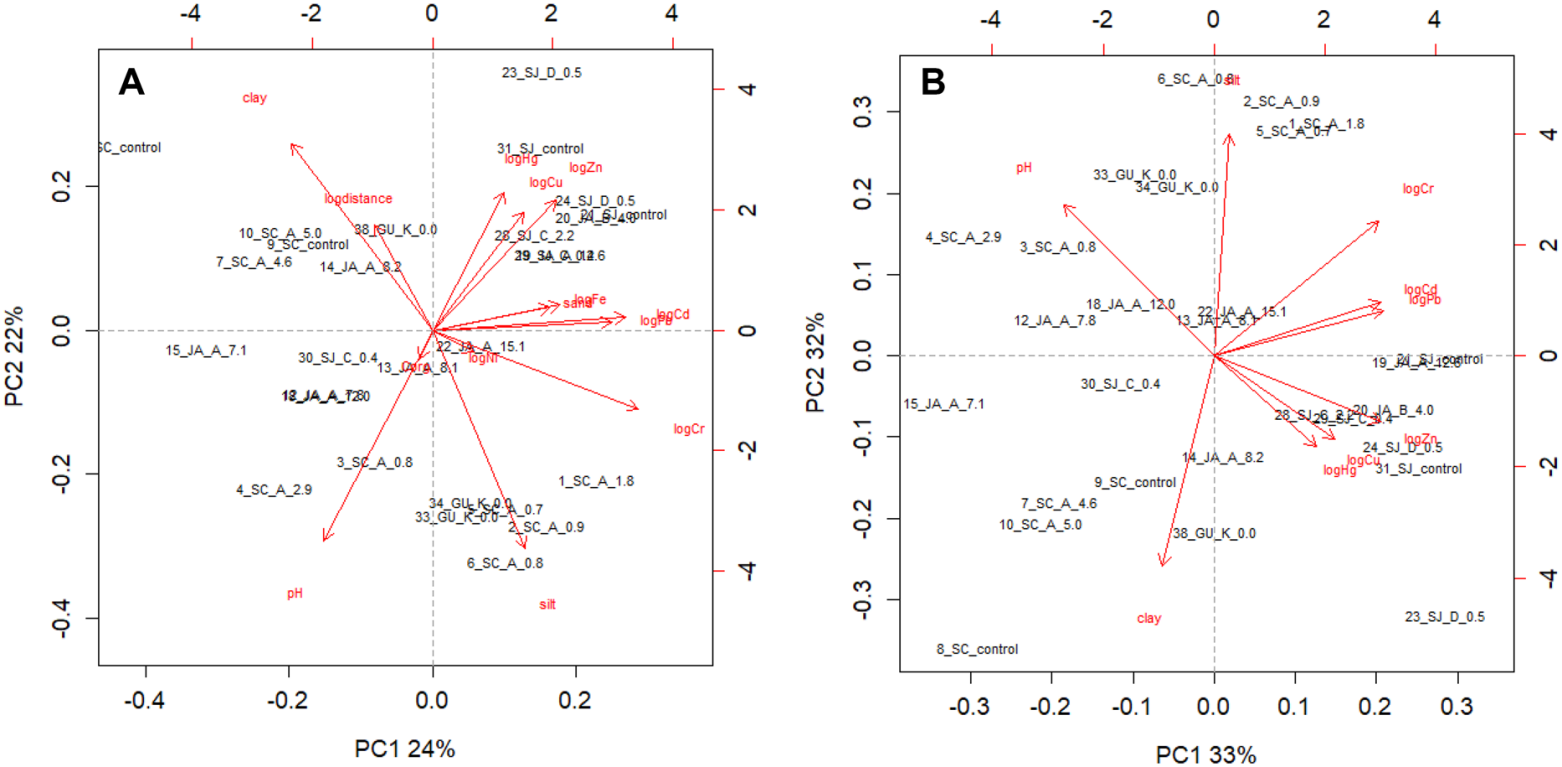
Biplot of the principal component analysis (PCA) of all (14) variables (all heavy metals, sand, silt, clay, pH, organic carbon (C_org_), and Fe) is shown in panel (**A**) and of the optimised PCA with increased variance and less variables (no Ni, Fe, C_org_, sand, and distance) is depicted in panel (**B**). Arrows are variables and grey names are the sites or observations. Principal component (PC) 1 of the optimised PCA (biplot B) explains 33% of the variation and PC2 32%. The observation names are a composite of site numbers, municipalities, the potential pollution source and the distance of the site to it in kilometres (Table S3). Abbreviations are Santa Cruz del Norte (SC), Jaruco (JA), San José de las Lajas (SJ), and Güines (GU). Letters indicate thermoelectric power plant (**A**), zeolite production plant (**B**), asphalt factory (**C**), cable industry (**D**), rubber manufactory (**E**), waste incineration (**F**, Table S3), and traffic/roads (**K**). Control sites (e.g. 31_SJ_control) are assumed not to be influenced by a pollution source. Please note that there are only 28 observations due to missing values in the data set (Table S6)

**Fig. 3:**
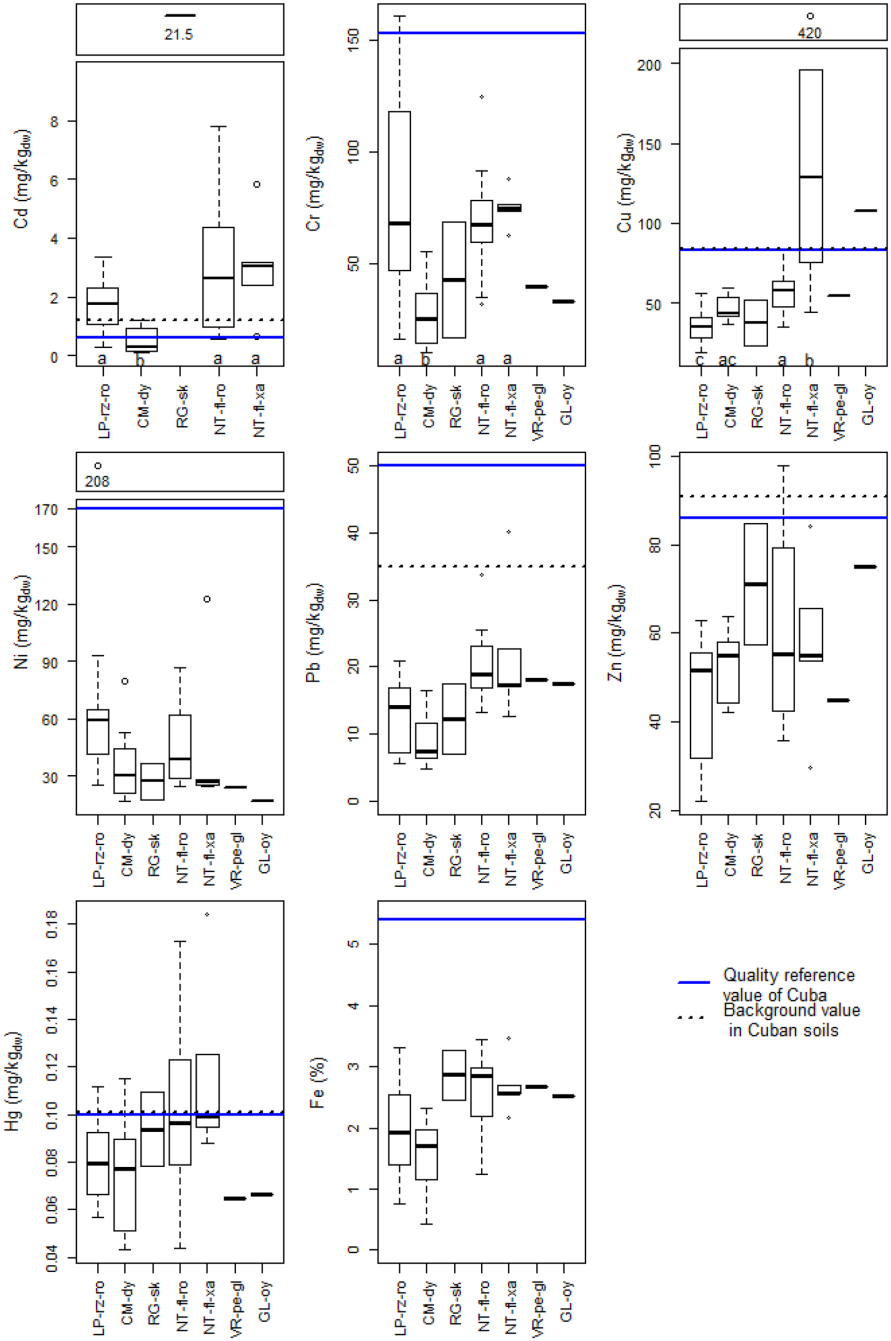
Heavy metals in the soils of Mayabeque, Cuba, divided into different soil types. The boxes represent the 25^th^ to 75^th^ percentiles, the whiskers are the 10^th^ and 90^th^ percentiles, the dots outliers, and the bold black line in the box indicates the median of the respective concentrations. Sample numbers are *n* = 11 for rhodic, rendzic leptosols (LP-rz-ro), *n* = 7 for dystric cambisols (CM-dy), *n* = 2 for skeletic regosols (RG-sk), *n* = 12 for rhodic, ferralic nitisols (NT-fl-ro), *n* = 5 for xanthic, ferralic nitisols (NT-fl-xa), *n* = 1 for gleyic, pellic vertisol (VR-pe-gl), and *n* = 1 for oxygleyic gleysol. Because of no detects for Cd, VR-pe-gl, and GL-oy are not displayed in the Cd-panel. Letters in the boxplots (Cd, Cr, Cu) specify a significant influence of the factor soil type according to the ANOVA model (Table S5). If that was the case, a pairwise comparison according to Bonferroni was performed and different letters indicated the difference within the group factor. The soils RG-sk, VR-pe-gl, and GL-oy were omitted due to the low sample number in the pairwise comparison and did therefore not receive a letter. The blue solid lines indicate quality reference value in Cuba (Rodríguez et al., 2015) and black dotted lines background values in Cuba (Rodríguez et al., 2015). The background value of Cr is 463 mg/kg_dw_ and of Ni 294 mg/kg_dw_

**Fig. 4:**
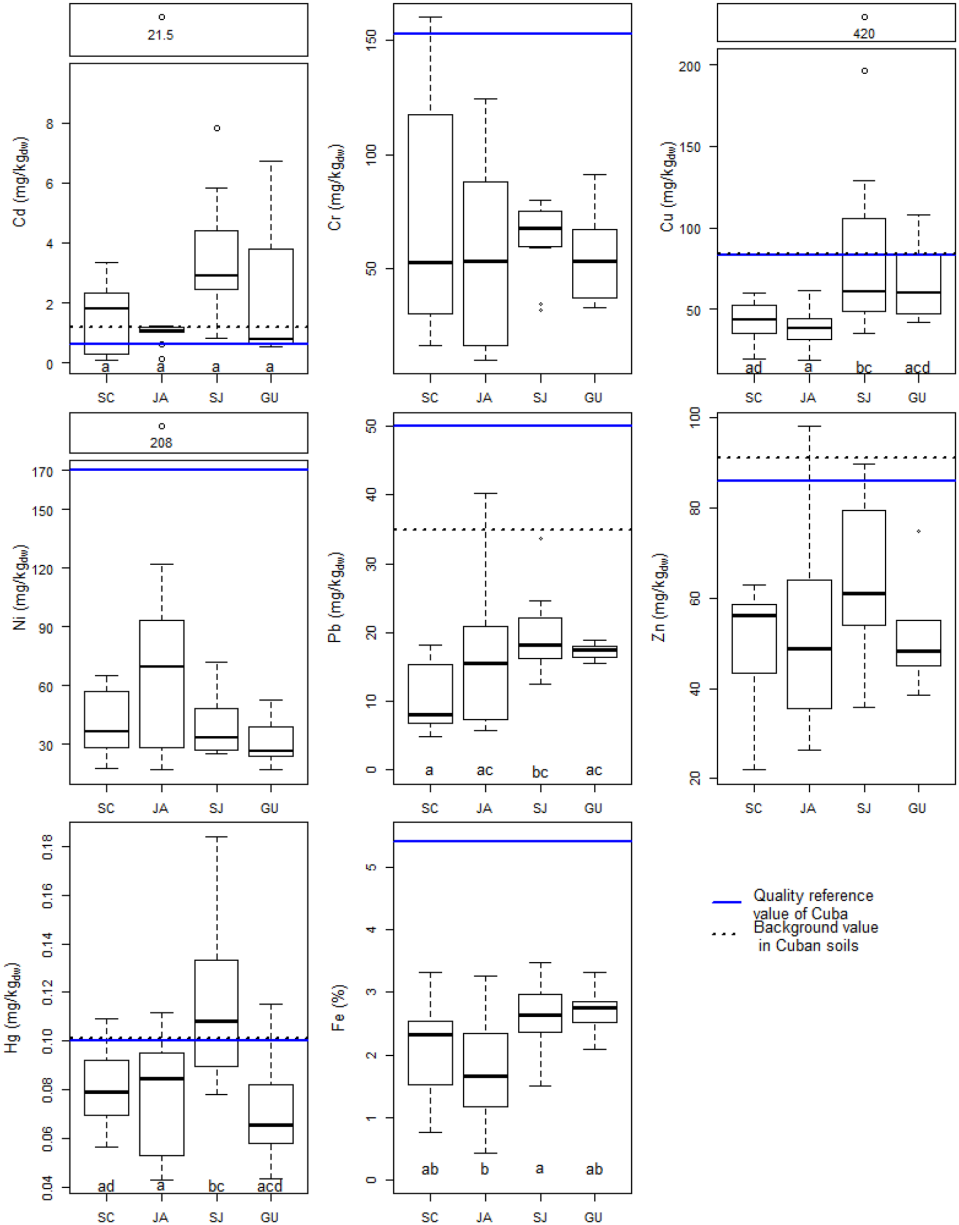
Heavy metals in the soils of Mayabeque, Cuba, divided into different municipalities (Santa Cruz = SC, Jaruco = JA, San Jose = SJ, Guines = GU). The boxes represent the 25^th^ to 75^th^ percentiles, the whiskers are the 10^th^ and 90^th^ percentiles, and the dots are outliers. Sample numbers of the municipalities were SC = 11, JA = 10, SJ = 12, and GU = 6. The bold black line in the box represents the median of the respective concentrations of the municipality; letters in the boxplots (Cd, Cu, Pb, Hg) specify a significant influence of the factor municipality according to the ANOVA model (Table S5). If that was the case, a pairwise comparison according to Bonferroni was performed and different letters indicated the difference within the group factor. The blue solid lines indicate quality reference value in Cuba (Rodríguez et al., 2015) and black dotted lines background values in Cuba (Rodríguez et al., 2015). The average background value of Cr is 463 mg/kg_dw_ and of Ni 294 mg/kg_dw_

All data are presented on a dry weight basis. All statistical analyses were run in R version 3.4.3 (2017–11-30). A PCA with numeric variables was run to discern anthropogenic inputs of heavy metals from pedogenic origins and the first two principal components (PC) were depicted in the biplot (Fig. 2, additional information about biplot and PCA is provided in chapters S2 and S3 of the ESI, respectively). A Pearson correlation analysis of heavy metal and Fe concentrations, soil properties and distance to potential pollution/contamination sources (Table S9) identifies important relationships between variables and helps interpret the biplot. A full ANOVA model with all factors including their interactions and combinations of it was run according to the following equation:

1 $${y}_{\text{ijkl}}=\mu +{\alpha }_{\text{i}}+{\beta }_{\text{j}}+{Y}_{\text{k}}+{\left(\alpha \beta Y\right)}_{\text{ijk}}+{\varepsilon }_{\text{ijkl}}$$

where *y* represents the dependent variable that are heavy metals (including Fe), *α* (municipality), *β* (soil type), and *γ* (land use) are the independent variables or factors, *µ* is the mean and *ε* the residue. The null hypotheses (H_0_) were that the groups of a factor had no influence on heavy metal concentrations, for instance for municipality H_0_: *α*_SC_ = *α*_JA_ = *α*_SJ_ = *α*_GU_. The ANOVA model is explained in detail in Table S5. Factors were considered significant, if H_0_ could be rejected on the 5% level or the *p*-value was ≤ 0.05. The concentrations were logarithmised to achieve normally distributed residuals of the model. The residuals were also checked for homoscedasticity due to unequal group sizes. Both conditions were fulfilled for all heavy metals. If a factor had a significant influence, a pairwise comparison according to Bonferroni was performed and the significant differences within the group indicated with different letters (Figs. 3 and 4).

**Fig. 5:**
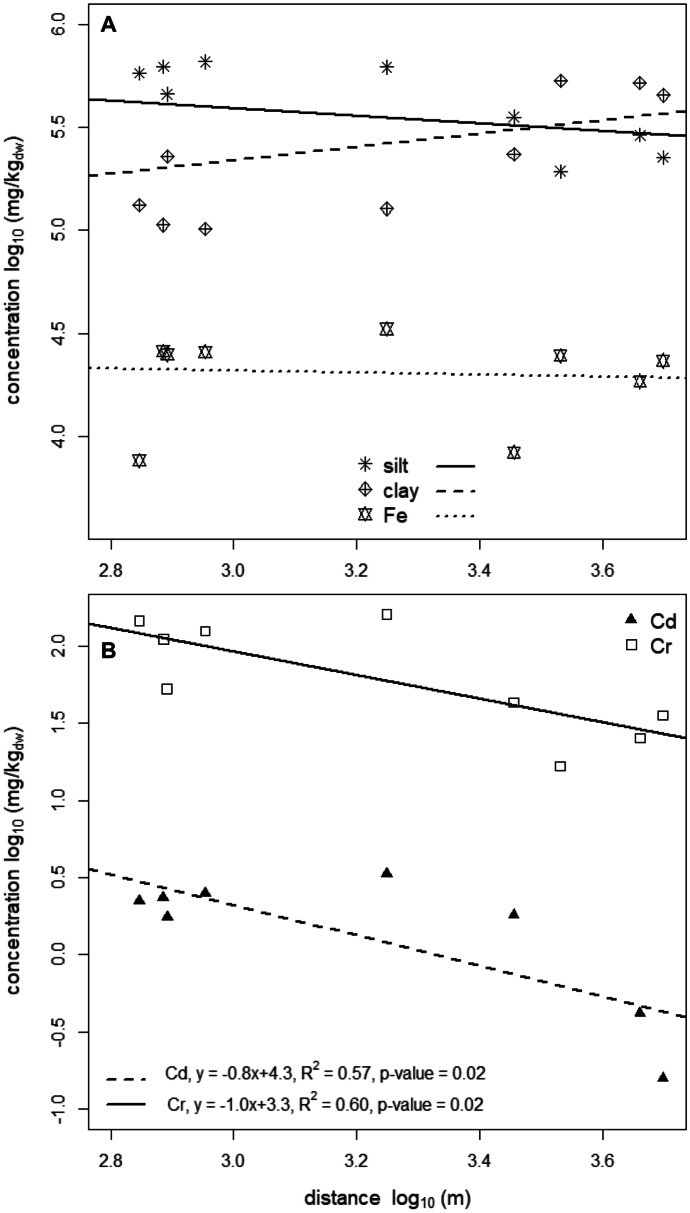
Silt, clay, and Fe contents (panel **A**) vs. distance to the thermoelectric power plant ( source A, Fig. 1). Linear regressions lines of the soil properties to distance are drawn but the evaluations were not significant. Concentration gradients (panel **B**) of cadmium (Cd, black triangles, *n* = 8) and chromium (Cr, white squares, *n* = 9) at different sites and distances from the thermoelectric power plant located in SC, Mayabeque, Cuba. Linear regression results are plotted into the graph as lines and equations and were both significant

Iron contents were opposed to heavy metal concentrations in scatter plots and compared with the linear regression models by Rodríguez et al. (2015) (Fig. S1). According to them, heavy metal concentrations in this study above the predicted ones would indicate an anthropogenic influence. Finally, a linear regression with Cd, Cr, Cu Ni, Pb, Zn, and Fe, each, as dependent variables and the distance to the most probable pollution sources thermoelectric power plant (Cd, Cr), asphalt factory (Cd, Cr, Cu Ni, Pb, Zn) and cable industry (Cu) was run to discern a pollution gradient of different sampling sites (Fig. 5).

## Results and discussion

Data are presented and interpreted from general to specific. In detail, we describe and compare heavy metal concentrations with literature and depict them in biplots originating from the PCA, prior to hypothesis-driven statistical analyses (ANOVA, Fe regression model based on Hamon et al. (2004) and Rodríguez et al. (2015), linear regression of heavy metal concentrations and gradients by distance). Table 1 summarises the results of the different evaluations. Statistically significant anthropogenic and geogenic influences are presented in capital letters “A” and “G” (and combinations of it for interactions), respectively, and those that were tentative/potentially indicative, but could not be tested statistically in “a” and “g” (and combinations of it if the source was both).

**Table 1:** Summary of literature and statistical analysis for the interpretation of cadmium (Cd), chromium (Cr), copper (Cu), nickel (Ni), lead (Pb), zinc (Zn), and mercury (Hg) concentrations in 39 soils of Mayabeque, Cuba. Statistically significant influences of anthropogenic sources received an “A” in the table, those under significant geogenic influence a “G”, and those that could not be tested or were evaluated qualitatively an “a” or “g”. If an interaction of the ANOVA was significant the entry received the combination of the significance code “AG” or “GA”. Dashes in the table indicate not measured

	**Cd**	**Cr**	**Cu**	**Ni**	**Pb**	**Zn**	**Hg**
a)**Comparison with literature**							
Median concentration > median concentration of pasture soil of Mayabeque and Artemisa by Amaral et al. (2013)							-
Median concentration > mean background concentration by Rodríguez et al. (2015)	g						
Median concentration > world crustal average concentration by Kabata-Pendias (2010)	g			g			
Median concentration > medians for soils of Parana State, Brazil, by Licht (2005)	ag						ag
b)**PCA qualitative influences**							
SJ has high concentrations of (PC1) → covered by the ANOVA with the factor municipality	-	-	-	-	-	-	-
Silt (PC2)		g					
Clay (PC2)		g					
pH (PC2)			g			g	
c)**ANOVA**							
Soil type	G	G	G				
Municipality	A		A		A		A
Land use							
Municipality * soil type		GA					AG
Municipality * land use							
Soil type * land use							
Municipality * soil type * land use	GA	GA					
d)**Fe enrichment factor**							
Concentration > Fe enrichment factor by Rodríguez et al. (2015)	a	a	a		a		a
e)**Linear regression of point source and distance**							
Thermoelectric power plant (A)	A	A					
f)**Potential point source of qualitative influence**							
Zeolite production (B)	-	-	-	-	-	-	-
Asphalt factory (C)							
Cable industry (D)			a				
Rubber factory (E)						a	
Waste incineration (F)						a	a
Textile production in Jaruco							
Agriculture in Güines							
Traffic/roads in Güines							
**Summary of influences**							
Statistically significant (all capital letters)	4	4	2	0	1	0	2
Geogenic influence (all g/G entries)	5	5	2	1	0	1	2
Anthropogenic influence (all a/A entries)	5	4	3	0	2	2	5
**Total entries (a–f)**							
	8	7	5	1	2	3	5

### Heavy metal concentrations measured and compared to literature

Individual soil concentrations of heavy metals are presented in Table S6. Median and concentration ranges (min–max) were for Cd 1.8 (0.1–21.6), Cr 60.3 (10.0–160), Cu 48.1 (18.6–420), Ni 36.2 (16.6–208), Pb 16.7 (4.9–40.2), Zn 55.0 (21.9–98.1), and Hg 0.09 (0.04–0.2) mg/kg (Table S7). Data is further presented and grouped according to soil types and municipalities in Figs. 3 and 4, respectively (for discussion, see below).

Chromium, Ni, Pb, and Zn concentrations were lower, and those of Cd and Cu were both spanning over a wider range but were mainly at the lower end compared to an earlier Cuban study that focused on pasture in the same province (Amaral et al., 2013). Chromium, Ni, Pb, and Zn in our study were in most samples also below the background values by Rodríguez et al. (2015), who quantified heavy metal contents in soil over the whole Cuban island. Some Cd and Cu as well as Hg concentrations were above these concentrations, though (Fig. 3, black dotted line, Table S7). Our data were mostly in the range or even lower compared to crustal (geological samples rather than top- or surface soil samples) average concentrations of Kabata-Pendias (2010) except for Cd and Ni. Cadmium and Hg in soils of this study were also higher than median concentrations for soils of the Paraná State in Brazil (Licht, 2005) (Table S7). The results of this section are summarised in Table 1, a.

### Data evaluation by statistical analyses and modelling

#### Heavy metal concentrations analysed descriptively by means of PCA

A PCA is an integral evaluation of all data with the aim to gain an overview of the dataset by reducing the variables and concomitantly keeping the variance (ESI, chapter S2). This is done in multiple steps. The first PCA was run with all (14) numeric variables (Cd, Cr, Cu, Ni, Pb, Zn, Hg, pH, distance to pollution source, C_org_, sand, silt, clay, and Fe) and is shown in the first biplot (Fig. 2A; how to read a biplot is explained in the ESI, chapter S3). Principal component 1 and PC2 in Fig. 2A have a variance of 46%, which means that less than half of the variance in the data set is represented with all numeric variables. However, both biplots (Fig. 2A, B) point, which is underlined by the correlation matrix (Table S9), to rather strong relationships between Cr and silt (*r* = 0.53), Cr and clay (*r* =  −0.61), Cr and Pb (*r* = 0.60), Cu and pH (*r* =  −0.51) and Zn and pH (*r* =  −0.52) compared to other correlation coefficients. This initial PCA was optimised to obtain a maximum variance with a minimum of variables (ESI, chapter S2). Therefore, C_org_, sand, distance, Fe, and Ni, variables that have a short arrow in the first biplot and did not contribute to an explanative orientation of the data, were removed. Thereby, the variance could be improved from former 46 to 65% (Fig. 2B).

The differentiation along PC1 and PC2 must not be interpreted in absolute values but in concert of all variables in the PCA. Principal component 1 discriminates the observations or soil samples (black labels in biplot B) according to the heavy metal concentrations with high contents on the right half of the biplot indicated by the direction of the corresponding arrows and low concentrations on the left half. (Please note that 11 samples are not depicted in Fig. 2A, B due to missing values.) Accordingly, Fig. 2B shows mostly sites from SJ (nomenclature of the observations explained in the figure caption) on the right half of the biplot as SJ has rather high Cd, Cu, Pb, Zn, and Hg concentrations in comparison to the other municipalities (Fig. 4). This finding might be due to the fact that most of the contamination or pollution sources are in SJ (Fig. 1). Principal component 2 discriminates according to texture. Sites with high silt (> 50%) are on the upper and those with a high clay (> 50%) content on the lower half of the biplot. As pointed out above, Cr contents are influenced by silt and clay. Furthermore, PC2 discriminates according to the pH with sites having a relatively low pH on the lower and locations with relatively high pH on the upper half of the biplot. Copper and Zn correlate the highest and negatively with pH (Table S9). Sites 23, 24 and 25 in SJ with soil pH around 5.5, the lowest of all soils, and the highest Cu concentrations (Table S6), probably influenced by the cable industry (Fig. 1D), provoke the leverage effect resulting in a diametrical position of the two variables in the biplot (Fig. 2B). Based on the discussion above, the sites are more or less evenly distributed in the biplot and no clusters, except maybe SJ observations on the right half of the biplot (Fig. 2B), are discernible. This points towards no clear-cut and prevailing influence of the observations (samples), for instance sites near a point source, on heavy metal concentrations/variables. The results of this section are summarised in Table 1, b.

#### Hypothesis-driven heavy metal concentration evaluations based on an ANOVA model—soil type

It is known from literature that elevated soil concentrations of heavy metals originate from the soil type and geogenic processes that contribute to soil type formation. For instance, 70% of Cuban soils are developed on sedimentary limestone and many of them are influenced by zones of ultramafic rocks that extend over Cuba and are characterised by, for instance, high contents of Cr and Ni (Camacho & Paulín, 1983; Cárdenas et al., 1986; Ruiz & Pérez, 1984). Therefore, the influence of soil type was tested in the ANOVA (incl. Fe), together with the other variables municipality and land use and all interactions (Eq. 1, Table S5). Soil type significantly influenced Cd, Cr, and Cu (Fig. 3, Table 1, c), which is in line with Amaral et al. (2013) and Rodríguez et al. (2015). The dystric cambisols (CM-dy) showed significant lower concentrations than the leptosols (LP) or nitisols (NT) for Cd and Cr (Fig. 3). Copper concentrations were also significantly lower in CM-dy than in xanthic ferralic NT (NT-fl-xa). A pedogenic origin probably also holds for site 20 showing the maximum Cd concentration in our study (21.5 mg/kg; Fig. 2, Table S6). This forest site was originally chosen as control site in our study. Its soil type, a skeletic regosole (RG-sk), is probably the reason for the high Cd concentration (Tables S3 and S6). Utermann et al. (2006) reported high Cd concentrations in poorly developed soils with high gravel contents and found similar Cd concentrations between 3 and 16 mg/kg in German forest soils. No soil type dependence was observed for any of the other observed (heavy) metals.

#### Hypothesis-driven heavy metal concentration evaluations based on an ANOVA model—municipality

Concerning the anthropogenic impact, the ANOVA model (Table S5) showed significant influences of the municipality on Cd, Cu, Pb, and Hg (Fig. 4, Table 1, c). Although the influence of the municipality was significant on Cd, the pairwise comparison did not show any significant difference in Fig. 4. Most pollution sources, i.e. the asphalt factory (C), the cable (D) and rubber (E) industry, and the waste incineration (F), are located in SJ (Fig. 1), which might be reflected in SJ having significantly higher Hg and Cu concentrations than JA and significantly higher Cu, Pb and Hg concentrations than SC (Fig. 4, Table S5). Interestingly, Fe itself in the ANOVA is significantly influenced by the factor municipality, which contributes to the difficulty in clearly distinguishing anthropogenic from pedogenic influences. Jaruco has significantly lower Fe concentrations than SJ and this behaviour is reflected in the lower concentrations of Cu and Hg but not for Cd and Pb concentrations (Fig. 4). Hence, the Fe impact does not seem to be systematic, which is why all combinations of factors were tested in the ANOVA as interactions too.

#### Hypothesis-driven heavy metal concentration evaluations based on an ANOVA model—land use

Land use (Fig. S2) alone did not have any significant influence on heavy metal concentrations but in combination with soil type and municipality for Cd and Cr and Cr and Hg showed significant interactions of soil type and municipality (Table 1, c, Table S5). The latter two interactions were plotted (Figs. S3 and S4). Generally, NT-fl-ro soils in GU had lower mean Cr concentrations in GU than in JA, which again had lower Cr concentrations in LP-rz-ro than SC, and if the soil was CM-dy, they were the lowest in JA and SC, while SJ only had NT-fl-xa and NT-fl-ro soils (Fig. S3). Mercury showed the highest mean concentrations in NT-fl-ro and NT-fl-xa soils of SJ, SC had higher mean Hg concentrations in CM-dy than in LP-rz-ro and JA had all soil types and means range from < 0.06 to > 0.1 mg/kg. Güines mainly had NT-fl-ro soils where Hg concentrations were around 0.06 mg/kg (Fig. S4). Nickel and Zn were not significantly influenced of any factor (Table S5).

#### Hypothesis-driven heavy metal concentration evaluations using a linear regression model based on iron contents in soil

Hamon et al. (2004) studied about 750 soils over the world and introduced a linear regression model, based on which heavy metal concentrations can be predicted by Fe concentrations. Rodríguez et al. (2015) studied the influence of Fe oxides on element background concentrations in soils with little anthropogenic influence in Cuba. They found highly significant and positive correlations between heavy metals and Fe. In contrast to their findings (*R*^2^ between Cd, Pb, Zn, Ni, Cr, Hg and Fe around 0.9 and for Cu = 0.65), correlation coefficients in this study were only ≤ 0.16 (Table S8 with heavy metal and Fe concentrations from Table S6). Moreover, concentrations of Cd, Cr, Cu, Pb and Hg were on average a factor of 19, 3, 2, 134, and 4, respectively, above predictions based on the linear Fe concentration correlation model (Fig. S1). This points to the presence of heavy metals in Cuban soils beyond geogenic backgrounds, possibly caused by anthropogenic emissions. However, such an interpretation requires caution, because relationships between heavy metals and Fe heavily depend on the weathering degree of soils, such that generic models might not be appropriate (Mikkonen et al., 2018). Moreover, these outcomes also depend on the type of assessment. If the ratio of the heavy metal over the Fe concentration of the sample is built and put over the same ratio of a reference soil, in our case Rodríguez et al. (2015), the enrichment factor (EF) gives an indication whether the soil is anthropogenically influenced or even polluted (Rizo et al., 2011). The EF calculations draw a different picture than Fig. S1. Only about a quarter of the Hg concentrations is moderately enriched, a few Zn concentrations, and the site with the elevated Ni content (Figs. 2 and 3). The results of the linear regression are summarised in Table 1, d.

#### Hypothesis-driven heavy metal concentration evaluations based on a linear regression models and potential influence of anthropogenic point sources

Figure 5 shows potential and significant relationships between concentrations of Cr and Cd in soils with distance to a thermoelectric power plant (sites 1–7, 10–15, 18, 19, 22; Tables S3 and S11 (Hämmann & Desaules, 2003)). Zinc and Pb (Hämmann & Desaules, 2003) were also related with distance, but the regression was not significant. To rule out any incidental influence of soil properties such as silt, clay (see above, as well as Facchinelli et al., 2001; Liu et al., 2011; Schulin et al., 2007; Shan et al., 2013) or Fe (see above) on the observed concentration gradients, correlations between these soil properties with distance were also tested, but proofed non-existing (Fig. 5). Hence, Cd and Cr concentration gradients observed over distance could indeed be influenced by the thermoelectric power plant (Table 1, e). (However, the significant influence of the thermoelectric power plant on Cr and Cd for instance was not visible nor the influence of other point sources in the PCA. As the PCA includes much more data than the 1:1 opposition in the linear regression, it probably reflects a more realistic picture than the regression.)

The distance of sites 23–25 to the cable industry (Table S3, Fig. 1D) and the Cu concentrations were fed into a linear regression model too but the relation turned out to be non-significant. However, Fig. 2B of the PCA suggests this influence by the closeness of site 23 in the projection and site 24 at the arrowhead of Cu (site 25 is not plotted due to missing values). Therefore, the cable industry and Cu received an entry in Table 1, f. Also the distance to the asphalt factory (sites 27–30, Fig. 1C) and concentrations of Cd, Cr, Cu, Ni, Pb, and Zn that can be emitted, amongst others, by this production (Ilechukwu et al., 2021) were put into a linear regression but was not significant. The asphalt factory did not raise local heavy metal concentrations either, which is why it had no entry in Table 1, f.

#### Potential influence of other anthropogenic point sources

Textile production in JA could lead to emission of Cr and Cu (Table S10; Hämmann & Desaules, 2003). However, this influence was not visible and Cr and Cu show comparable concentrations in JA, SC and GU (Fig. 4). In GU, agriculture should prevail where Pb and Cu from intensive cultivation (Table S10) should be seen, but this was not the case (Fig. 4). Mercury in soil can originate from manufactures of cement and metals, gold extraction, and caustic soda productions and from waste incinerations (Clifford et al., 2010). Indeed, point source F, the waste incineration (Table S3, site 32), could have contributed to the 0.14 mg/kg Hg and also to 77.6 mg/kg Zn (Tables S6 and S10) in this sample as these concentrations are on the upper end of the boxplots (Fig. 3). Sites 26 (0.18 mg/kg) and 21 (0.17 mg/kg; Table S6) had the highest Hg concentrations but could not be attributed to any source listed here. Site 26 is very close to the rubber manufactory (Fig. 1E, 0.4 km, Table S3). However, rubber production usually does not co-emit Hg (Clifford et al., 2010). The elevated concentration of Hg at site 21 (0.17 mg/kg), cropland, could be due to impurities in fertilisers (Mclaughlin et al., 1996). Though this is a plausible reason for site 21, it cannot explain the concentration at site 26 (0.18 mg/kg), which is pasture and therefore extensively managed. However, it is also possible that site 26 was cropland in the past.

Ma and Hooda (2010) indicate heavy industry and major roads as point sources of Ni but this influence could not be confirmed in this study. Nickel was also removed from the PCA (see below) due to bad representativeness and could neither be attributed to the thermoelectric power plant (Table S10) nor to any other pollution source. However, Ni is, together with Zn, the element that overlapped the most with iron predictions (Fig. S1). Hence, the factors to explain Ni concentrations are probably others than the ones investigated here.

According to Chaney (2010), fertilisers, fungicides and rubber industry contain or use Zn. Indeed, the rubber factory (E, site 26) in SJ could have contributed to the Zn concentration at site 26 (84.3 mg/kg only 0.4 km away). Traffic could have contributed to the Pb (Hough, 2010) but also to Zn (Table S10; Hämmann & Desaules, 2003) concentrations at sites 33–38 in GU with leaded petrol consumption and with abraded tires containing Pb, Zn, Cd, and Cu (Wang et al., 2021). However, the car frequency in Cuba is probably too low to be of discernible influence.

No information was found that the production of zeolite (sites 16, 17, 20, Fig. 1B) should emit any heavy metals. To the contrary, zeolite is a highly sorptive aluminosilicate (Pahlavanzadeh & Motamedi, 2020) that is often used to bind heavy metals for remediation purposes for instance in soils (Ma et al., 2022) or wastewaters (Elboughdiri, 2020; Pahlavanzadeh & Motamedi, 2020). The results of this section are summarised in Table 1, f.

#### Summary

In summary, our data evaluation revealed no unequivocal indication whether heavy metal concentrations in Mayabeque, Cuba, were overwhelmingly influenced geo- or anthropogenically. Linear regression models based on Fe concentrations suggested additional heavy metal sources beyond a geogenic background, but are probably not very trustworthy when applied generically (Mikkonen et al., 2018). Soil types and municipality explained the presence of a heavy metals occasionally only by ANOVA. Soil pH and texture showed correlations with some heavy metals. Presumed point sources show a limited effect on the heavy metal concentrations in a few specific cases at best. The original attempt of the PCA to relate potential contamination and/or pollution sources to individual sites failed as the variable distance did not contribute to the overall variance and because the contamination and/or pollution sources were probably too few and the influence too weak to be seen in a multidimensional system as the biplot of the PCA.

Table 1 provides a compilation of the outcomes of all data evaluations. With eight and seven entries, Cd and Cr, respectively, were the heavy metals for which most information could be extracted from the raw data. Nickel and Pb were the least tangible ones in this respect. Furthermore, Cd and Cr had the highest number of significant entries, four, each, while Cu and Hg had two, each, Pb one and Ni and Zn showed only a qualitative influence. While Cd, Cr and Ni seemed to be rather geogenically influenced, Cu, Pb, Zn, and Hg concentrations were rather provoked by human activities. Consequences for heavy metal concentrations on regulation are elaborated in the next section.

### Heavy metal concentrations in light of different environmental quality standards

An official regulation for heavy metal concentrations in soils does not exist in Cuba. However, the study of Rodríguez et al. (2015) and Amaral et al. (2013) prepare, together with the present study, the way for Cuban legislation to establish threshold values for heavy metals. The QRV for Cuban soils of Rodríguez et al. (2015) (Table S7) are based on surface soils of the country under natural conditions with presumably little anthropogenic interference. This can be observed in the QRV concentrations of Cd, Ni, and Pb that are factors higher than crustal average concentrations of the world (Kabata-Pendias, 2010). Cuban QRV of Cd, Ni, Pb, and Hg are also factors higher than median concentrations for soils of Paraná State, Brazil (Licht, 2005) (Table S7).

Regulations from other countries, such as Switzerland and Brazil (Table S7), were also considered to interpret the concentrations of heavy metals in this study. The Swiss threshold values (guide and clean-up values VBBo (1998)) were used as a reference as they are defined for the extraction method used in this study and adequacy of the results in tropical soils is outlined in the “Quality control” section. If guide values are surpassed, soils can be used without limitations according to Swiss legislation, as they are still considered of no health concern. However, the soil has to be monitored and care has to be taken that the concentration of the respective metal does not rise further.

The investigation values (IV) of Brazil (Table S7; CONAMA, 2009), another expression for environmental quality standard and similar to the Swiss clean-up label, are probably more realistic to compare with Cuban soils. According to this regulation, concentrations of heavy metals in soil above the IV pose a risk to human health and development of organisms, and stipulate specific actions, such as restrictions on soil use, or soil remediation.

Most soil concentrations of Cr, Ni, Pb, and Zn were below QRV in the province of Mayabeque (Figs. 3 and 4 blue, solid line). Mercury concentrations were above the QRV in about a third (11 out of 39) of the samples, which indicates that the Hg QRV of 0.1 mg/kg is rather low in comparison to the Swiss (0.5 mg/kg) and Brazilian (12 mg/kg) threshold values (Table S7). Copper concentrations were generally below the QRV, with the exception of the sites around the cable industry (sites 23–25) that are all (incl. 22 and 26) located on the NT-fl-ro where the soil type had a significant influence on Cu too. Cadmium concentrations were above the QRV for most sites (29 of 39), which might be, especially in SJ, influenced by the soil types LP and NT (Fig. 3).

The concentrations of Pb, Zn and Hg were below the Swiss guide and the Brazilian IV at all sites (Table S7). Hence, there was no problematic contamination and/or pollution in terms of Swiss and Brazilian environmental quality standards of these metals in Cuban soils. Swiss clean-up values existing for Cd, Cu, Pb, and Zn (Table S7) were never exceeded in any of the Cuban soils. Though, Cd, Cr, Cu, and Ni exceeded the Swiss guide values at different sites. Concentrations of Ni were above the Brazilian IV at 21 sites (54%), of Cd at 10 sites (26%) and of Cr and Cu at one site (Tables S6 and S7). In summary, Cd and Ni were elements with elevated concentrations in the soils of this study exceeding Brazilian IV representing environmentally adequate quality standards for tropical soils. These high concentrations seem to be anthropogenically as well as pedogenically influenced, and future Cuban threshold values might need to be higher than current QRV, to avoid an enforcement dilemma.

## Conclusions

Heavy metal concentrations in Mayabeque soils were mostly below the Cuban QRV. While Cd, Cr and Ni concentrations were rather pedogenically influenced, Cu, Pb, Zn, and Hg contents were rather anthropogenically driven. However, when evaluated statistically, Cd and Cr showed most times a significant influence, while, for instance, Ni and Zn showed none. Hence, the allocation of heavy metal concentrations to pedogenic or anthropogenic contamination or pollution sources is tentative and needs further investigations. The evaluations are in line with the description of Mayabeque being mainly influenced by agriculture of which half of it is extensively managed. However, Cd together with some Cu concentrations exceeded not only QRV but Cd and occasionally Ni surpassed the Brazilian IV. These sites pose a potential risk for human and environmental health according to the Brazilian IV.

To protect Cuban soils from heavy metal contamination, QRV must be further developed. On the one hand, QRV might be adapted, as, for instance, the values for Cd and Cu should be raised after extended Fe calibration concentrations due to the high pedogenic baseline. On the other hand, soil monitoring should be extended and executed regularly as already suggested in Sosa et al. (2017, 2019). A long-term monitoring network allows the researchers to report to the CITMA on provincial and national levels. The authority can develop sound threshold values protecting human and environmental health and allowing interventions if necessary.

## Supplementary Information

Below is the link to the electronic supplementary material.

Supplementary file1 (DOC 555 KB)
