# Supplementary material for: Heavy metals in soils of Mayabeque, Cuba: multifaceted and hardly discernable contributions from pedogenic and anthropogenic sources
Source: Environ Monit Assess. 2022 May 20;194(6):441. doi: 10.1007/s10661-022-10097-6 (PMC9123067; doi:10.1007/s10661-022-10097-6)
Supplement: Supplementary file 1 — Supplementary file1 (DOC 555 KB) [file 10661_2022_10097_MOESM1_ESM.doc]

**Supplementary Information**

**Heavy metals in soils of Mayabeque, Cuba: multifaceted and hardly discernable contributions from pedogenic and anthropogenic sources**

Dayana Sosa^a^, Isabel Hilber^b^, Diane Buerge-Weirich^b,†^, Roberto Faure^a^, Arturo Escobar^a,†^, and Thomas D. Bucheli^b^

^a^, Centro Nacional de Sanidad Agropecuaria (CENSA), Apartado 10, CP32700, San José de las Lajas, Mayabeque, Cuba

^b^, Environmental Analytics, Agroscope, Reckenholzstrasse 191, CH-8046 Zurich, Switzerland,

† Corresponding authors: escobar@censa.edu.cu, Tel +53 64 63024, Fax: +53 64 63897; diane.buerge@agroscope.admin.ch, Tel: +41 58 468 71 60, Fax: +41 58 468 72 01

## Soil classification

The soil types of the sampling sites were determined by Hernández-Jiménez et al. (2015) and Lopez-Kramer (2012) and then “translated” to the World Reference Base (WRB, Table S1) for soil resources 2014 (IUSS Working Group WRB, 2015) that is an international soil classification system for naming soils and creating legends for soil maps. Table S1 gives an overview of the first level WRB soils categorization that comprises 32 reference soil groups (RSG) and the second level classification consisting of principal and supplementary qualifier properties of soils. Principal qualifiers are regarded as being most significant for a further characterization of soils of the particular RSG. They are given in a ranked order. Supplementary qualifiers give some further details about the soil.

Table S1 provides the final soil type classified in Cuba by analytical data and translated into the WRB classification. Hence, some physico-chemical properties of the soils are implicit in the soil types, which is why they were not anymore analysed in this study.

| **Table S1: Evolution from Cuban soil types into the World Reference Base (WRB) for soil resources 2014 (IUSS Working Group WRB, 2015)**. | | |
| --- | --- | --- |
| **Soil type (Cuba)** | **Soil type translated in English** | **WRB soil types** |
| Rendzina Roja | Red Rendzine | Rhodic Rendzic Leptosols |
| Pardo Grisaceo | Greyish brown | Dystric Cambisol |
| Esquelético | Skeletal | Skeletic Regosols |
| Ferralítico Rojo | Red ferralitic | Rhodic Ferralic Nitisol |
| Ferralítico Amarillo | Yellow ferralitic | Xanthic Ferralic Nitisol |
| Oscuro plástico gley | Dark plastic gley | Gleyic Pellic Vertisol |
| Gley amarillo cuarsitico | Gley yellow quartzite | Oxygleyic Gleysols |

| **Table S2: Soil types in this study (last column) described according their reference soil group (RSG), management and use and qualifier properties. All soil type information in the table is from the World Reference Base (WRB) for soil resources 2014 (IUSS Working Group WRB, 2015).** | | | | |
| --- | --- | --- | --- | --- |
|  |  | **Definitions of qualifiers** | |  |
| **Reference soil group (RSG)** | **Management and use** | **Supplementary qualifier** | **Principal qualifier** | **Soil type in study** |
| **Leptosol** (LP): Connotation: Thin soils; from Greek *leptos*, thin. Parent material: Various kinds of continuous rock or of unconsolidated materials with less than 20 percent (by volume) fine earth. Profile development: LP have continuous rock at or very close to the surface or are extremely gravelly. LP in weathered calcareous material may have a mollic horizon. Environment: Mostly land at high or medium altitude and with strongly dissected topography. LP are found in all climate zones (many of them in hot or cold dry regions), in particular in strongly eroding areas. | LP have a resource potential for wet-season grazing and as forest land. LP to which the rendzic qualifier applies are planted with teak and mahogany in Southeast Asia; those in the temperate zone are under mainly deciduous mixed forest whereas acid LP are commonly under coniferous forest. Erosion is the greatest threat to LP areas, particularly in mountain regions in the temperate zones where high population pressure (tourism), overexploitation and increasing environmental pollution lead to deterioration of forests. LP on hill slopes are generally more fertile than their counterparts on more level land. One or a few good crops could perhaps be grown on such slopes but at the price of severe erosion. Steep slopes with thin and stony soils can be transformed into cultivable land through terracing, the removal of stones by hand and their use as terrace fronts. Agroforestry (a combination or rotation of arable crops and trees under strict control) holds promise but is still largely in an experimental stage. The excessive internal drainage and the shallow depth of many LP can cause drought even in a humid environment. | Rhodic (ro): (from Greek *rhodon*, rose): having between 25 and 150 cm of the soil surface, a layer ≥ 30 cm thick, that has, in ≥ 90% of its exposed area, a Munsell colour hue redder than 5YR moist, a value of < 4 moist, and a value dry, no more than one unit higher than the moist value (2: except Epi-). | Rendzic (rz) (from Polish *rzendzic*, to grate in contact with a plough blade): having a mollic horizon that contains or directly overlies *calcaric* material containing ≥ 40% calcium carbonate equivalent or that directly overlies calcareous rock containing ≥ 40% calcium carbonate equivalent (2: Ano- and Panto- only). | Rhodic Rendzic Leptosols  LP-rz-ro |
| **Cambisol** (CM): Connotation: Soils with at least the beginnings of horizon differentiation in the subsoil, evident from changes in structure, colour, clay content or carbonate content from Late Latin *cambiare*, to change.  Parent material: Medium and fine textured materials derived from a wide range of rocks.  Profile development: CM are characterized by slight or moderate weathering of parent material and by absence of appreciable quantities of illuviated clay, organic matter, Al and/or Fe compounds. CM also encompass soils that fail one or more characteristics diagnostic for other RSGs, incl. highly weathered ones.  Environment: Level to mountainous terrain in all climates; wide range of vegetation types. | CM generally make good agricultural land and are used intensively. CM with high base saturation in the temperate zone are among the most productive soils on earth. More acid CM, although less fertile, are used for mixed arable farming and as grazing and forest land. CM on steep slopes are best kept under forest; this is particularly true for CM in highlands. CM on irrigated alluvial plains in the dry zone are used intensively for production of food and oil crops. CM in undulating or hilly terrain are planted with a variety of annual and perennial crops or are used as grazing land. CM in the humid tropics are typically poor in nutrients but are still richer than associated *Acrisols* or *Ferralsols*, and they have a greater cation exchange capacity (CEC). CM with groundwater influence in alluvial plains are highly productive *paddy soils*. | Dystric (dy) (from Greek *dys*, bad, and *trophae*, food): having:   - in Histosols, a pH water < 5.5 in half or more of the part with organic material, within 100 cm of the soil surface - in other soils, an effective base saturation [exchangeable (Ca + Mg + K + Na)/exchangeable (Ca + Mg + K + Na + Al); exchangeable bases by 1 M NH_4_OAc (pH 7), exchangeable Al by 1 M KCl (unbuffered)] of < 50%:   - in half or more of the part between 20 and 100 cm from the mineral soil surface, or   - in half or more of the part between 20 cm from the mineral soil surface and continuous rock, technic hard material or a cemented or indurated layer starting > 25 cm from the mineral soil surface, or   - in a layer ≥ 5 cm thick, directly above continuous rock, technic hard material   - or a cemented or indurated layer starting ≤ 25 cm from the mineral soil surface. |  | Dystric Cambisol  CM-dy |
| **Regosol** (RG): Connotation: Weakly developed soils in unconsolidated material; from Greek *rhegos*,  blanket. Parent material: unconsolidated, generally fine-grained material. Profile development: No diagnostic horizons. Profile development is minimal because of young age and/or slow soil formation, e.g. because of aridity. Environment: All climate zones without permafrost and at all elevations. RG are particularly common in arid areas (incl. the dry tropics) and in mountain regions. | RG in desert areas have minimal agricultural significance. RG with rainfall of 500–1 000 mm/year need irrigation for satisfactory crop production. The low moisture holding capacity of these soils calls for frequent applications of irrigation water; sprinkler or trickle irrigation solves the problem but is rarely economic. Where rainfall exceeds 750 mm/year, the entire profile is raised to its water holding capacity early in the wet season; improvement of dry farming practices may then be a better investment than installation of costly irrigation facilities. Many RG are used for extensive grazing. RG on colluvial deposits in the loess belt of Europe and North America are mostly cultivated; they are planted with small grains, sugar beet and fruit trees. RG in mountainous regions are delicate and best left under forest. | Skeletic (sk) (from Greek *skeletos*, dried out): having ≥ 40% (by volume) coarse fragments averaged over a depth of 100 cm from the soil surface or to continuous rock, technic hard material or a cemented or indurated layer, whichever is shallower. |  | Skeletic Regosols  RG-sk |
| **Nitisol** (NT): Connotation: Deep, well-drained, red tropical soils with a clayey *nitic* horizon that has typical angular blocky structure breaking into polyhedral or flat-edged or nut-shaped elements with, in moist state, shiny aggregate faces; from Latin *nitidus*, shiny. Parent material: Finely textured weathering products of intermediate to basic parent rock, in some regions rejuvenated by recent admixtures of volcanic ash. Profile development: Red or reddish-brown clayey soils with a *nitic* subsurface horizon of high aggregate stability. The clay assemblage of Nitisols is dominated by kaolinite/(meta) halloysite. NT are rich in Fe and have little water-dispersible clay. Environment: NP are predominantly found on level to hilly land under tropical rainforest or savannah vegetation. | NT are among the most productive soils of the humid tropics. The deep and porous solum and the stable soil structure of NT permit deep rooting and make these soils quite resistant to erosion. The good workability of NT, their good internal drainage and fair water holding properties are complemented by chemical (fertility) properties that compare favorably with those of most other tropical soils. NT have relatively high contents of weathering minerals, and surface soils may contain several percent of organic matter, in particular under forest or tree crops. NT are planted with plantation crops, such as cocoa, coffee, rubber and pineapple and are also widely used for food crop production on smallholdings. High P sorption calls for application of P fertilizers, usually provided as slow-release, low-grade phosphate rock (several tonnes per hectare, with maintenance doses every few years) in combination with smaller applications of better soluble superphosphate for short-term response by the crop. | Rhodic (ro): (from Greek *rhodon*, rose): having between 25 and 150 cm of the soil surface, a layer ≥ 30 cm thick, that has, in ≥ 90% of its exposed area, a Munsell colour hue redder than 5YR moist, a value of < 4 moist, and a value dry, no more than one unit higher than the moist value (2: except Epi-). | Ferralic (fl) (from Latin *ferrum*, iron, and *alumen*, alum): having a ferralic horizon starting ≤ 150 cm of the soil surface. | Rhodic Ferralic Nitisol  NT-fl-ro |
|  |  | Xanthic (xa) (from Greek *xanthos*, yellow): having a ferralic horizon that has in a subhorizon ≥ 30 cm thick, and starting ≤ 75 cm of the upper limit of the ferralic horizon, in ≥ 90% of its exposed area, a Munsell colour hue of 7.5YR or yellower, a value of ≥ 4 and a chroma of ≥ 5, all moist. |  | Xanthic Ferralic Nitisol  NT-fl-xa |
| **Vertisol** (VR): Connotation: Churning, heavy clay soils from Latin *vertere*, to turn. Parent material: Sediments that contain a high proportion of swelling clays, or swelling clays produced by neoformation from rock weathering. Profile development: Alternate swelling and shrinking of expanding clays results in deep cracks in the dry season, and formation of slickensides and wedge-shaped structural elements in the subsurface soil. Shrink-swell behaviour may also cause gilgai microrelief to form, especially in drier climates. Environment: Depressions and level to undulating areas, mainly in tropical and subtropical, semi-arid to subhumid and humid climates with an alternation of distinct wet and dry seasons. The climax vegetation is savannah, natural grassland and/or woodland. | Large areas of VR in the semi-arid tropics are still unused or are used only for extensive grazing, wood chopping, charcoal burning and the like. These soils have considerable agricultural potential, but adapted management is a precondition for sustained production. The comparatively good chemical fertility and their occurrence on extensive level plains where reclamation and mechanical cultivation can be envisaged are assets of VR. Their physical soil characteristics, and notably their difficult water relations, cause management problems. Buildings and other structures on VR are at risk and engineers have to take special precautions to avoid damage. The agricultural uses of VR range from very extensive (grazing, collection of fuelwood and charcoal burning) through smallholder post-rainy season crop production (millet, sorghum, cotton and chickpeas) to small-scale (rice) and large-scale irrigated agriculture (cotton, wheat, barley, sorghum, chickpeas, flax, noug [*Guzotia abessynica*] and sugar cane). Cotton is known to perform well on VR, apparently because cotton has a vertical root system that is not damaged severely by cracking of the soil. Tree crops are generally less successful because tree roots find it difficult to establish themselves in the subsoil and are damaged as the soil shrinks and swells. Management practices for crop production should be directed primarily at water control in combination with conservation or improvement of soil fertility. The physical properties and the soil moisture regime of VR represent serious management constraints. The heavy soil texture and domination of expanding clay minerals result in a narrow soil moisture range between moisture stress and water excess. Tillage is hindered by stickiness when the soil is wet and hardness when it is dry. The susceptibility of VR to waterlogging may be the single most important factor that reduces the actual growing period. Excess water in the rainy season must be stored for post-rainy season use (water harvesting) on VR with very low infiltration rates. A compensation for the shrink–swell characteristics is the phenomenon of selfmulching that is common on many VR. Large clods produced by primary tillage break down with gradual drying into fine aggregates, which provide a passable seed bed with minimal effort. For the same reason, gully erosion on overgrazed VR is seldom severe because gully walls soon assume a shallow angle of repose, which allows grass to become re-established more readily. | Gleyic (gl) (from Russian *gley*, mucky soil mass): having a layer ≥ 25 cm thick, and starting ≤ 75 cm from the mineral soil surface, that has gleyic properties throughout and reducing conditions in some parts of every sublayer. | Pellic (pe) (from Greek *pellos*, dusty): having in the upper 30 cm of the soil a Munsell colour value of ≤ 3 and a chroma of ≤ 2, both moist (in Vertisols only). | Gleyic Pellic Vertisol  VR-pe-gl |
| **Gleysol** (GL): Connotation: Soils with clear signs of groundwater influence; from Russian *gley* (as soil  name introduced by G.N. Vysotskiy in 1905), mucky mass. Parent material: A wide range of unconsolidated materials, mainly fluvial, marine and lacustrine sediments. Profile development: Evidence of reduction processes with segregation of Fe compounds starting within 40 cm of the soil surface. Environment: Low positions in landscapes with high groundwater table, tidal areas, shallow lakes and sea shores. | For many GL, the main obstacle to utilization is the necessity to install a drainage system to lower the groundwater table. Adequately drained GL can be used for arable cropping, dairy farming and horticulture. Soil structure will be destroyed for a long time if soils are cultivated when too wet. Therefore, GL in depression areas with unsatisfactory possibilities to lower the groundwater table are best kept under a permanent grass cover or swamp forest. Liming of drained GL that are high in organic matter and/or of low pH value creates a better habitat for micro- and meso-organisms and enhances the rate of decomposition of soil organic matter (and the supply of plant nutrients). GL can be put under tree crops only after the water table has been lowered with deep drainage ditches. Alternatively, the trees are planted on ridges that alternate with shallow depressions in which rice is grown. This *sorjan* system is applied widely in tidal swamp areas with pyretic sediments in Southeast Asia. GL can be used for wetland rice cultivation where the climate is appropriate. GL with a thionic horizon or oxidized hypersulfidic material suffer from severe acidity and high levels of Al toxicity. Underwater and tidal GL are used for fishing or shrimp production. Many are left under natural conditions. Tidal lands that are strongly saline are best kept under mangroves or some other salt-tolerant vegetation. Such areas are ecologically valuable and can, with caution, be used for fishing, hunting, salt pans or woodcutting for charcoal or fuelwood. |  | Oxygleyic (oy) (from Greek *oxys*, sour, and Russian gley, mucky soil mass): not having, within ≤ 100 cm of the mineral soil surface, a layer that meets diagnostic criterion 1 of the gleyic properties (in Gleysols only). | Oxygleyic Gleysols  GL-oy |

| **Table S3. Characterization of soil samples gathered in the province of Mayabeque, Cuba.** | | | | | | | | |
| --- | --- | --- | --- | --- | --- | --- | --- | --- |
| **Site**  **Number** | **Munici­palities^a^** | **Lat (^o^)** | **Lon (^o^)** | **Altitude**  **(m a.s.l.)** | **Soil Type^b^** | **Land use^c^** | **Main Potential emission source^d^** | **Distance from main potential contamination/pollution source (km)** |
| 1 | SC | 23.14558 | -81.94994 | 92 | LP-rz-ro | Pasture | Thermoelectric power plant (A) | 1.8 |
| 2 | SC | 23.15347 | -81.95116 | 18 | LP-rz-ro | Crop | Thermoelectric power plant (A) | 0.9 |
| 3 | SC | 23.15450 | -81.95150 | 27 | LP-rz-ro | Pasture | Thermoelectric power plant (A) | 0.8 |
| 4 | SC | 23.14091 | -81.97133 | 106 | LP-rz-ro | Crop | Thermoelectric power plant (A) | 2.9 |
| 5 | SC | 23.15483 | -81.95326 | 112 | LP-rz-ro | Crop | Thermoelectric power plant (A) | 0.7 |
| 6 | SC | 23.15427 | -81.95291 | 107 | LP-rz-ro | Crop | Thermoelectric power plant (A) | 0.8 |
| 7 | SC | 23.11979 | -81.95266 | 90 | CM-dy | Pasture | Thermoelectric power plant (A) | 4.6 |
| 8 | SC | 23.12624 | -81.97238 | 49 | CM-dy | Crop | Negative control | - |
| 9 | SC | 23.15989 | -81.98292 | 29 | LP-rz-ro | Pasture | Negative control | - |
| 10 | SC | 23.13860 | -81.91181 | 37 | CM-dy | Pasture | Thermoelectric power plant (A) | 5.0 |
| 11 | SC | 23.13260 | -81.96625 | 60 | RG-sk | Pasture | Thermoelectric power plant (A) | 3.4 |
| 12 | JA | 23.09291 | -81.97457 | 121 | LP-rz-ro | Crop | Thermoelectric power plant (A) | 7.8 |
| 13 | JA | 23.09216 | -81.98100 | 115 | LP-rz-ro | Pasture | Thermoelectric power plant (A) | 8.1 |
| 14 | JA | 23.09176 | -81.98248 | 118 | LP-rz-ro | Pasture | Thermoelectric power plant (A) | 8.2 |
| 15 | JA | 23.09666 | -81.95633 | 126 | LP-rz-ro | Pasture | Thermoelectric power plant (A) | 7.1 |
| 16 | JA | 23.05300 | -82.03597 | 121 | CM-dy | Pasture | Zeolite production (B) | 0.6 |
| 17 | JA | 23.06790 | -82.05685 | 93 | CM-dy | Pasture | Zeolite production (B) | 3.3 |
| 18 | JA | 23.05968 | -81.91366 | 135 | CM-dy | Crop | Thermoelectric power plant (A) | 12.0 |
| 19 | JA | 23.04973 | -81.93001 | 115 | NT-fl-ro | Crop | Thermoelectric power plant (A) | 12.6 |
| 20 | JA | 23.04273 | -82.06844 | 266 | RG-sk | Forest | Zeolite production (B) | 4.0 |
| 21 | SJ | 22.98858 | -82.13999 | 133 | NT-fl-ro | Crop | Negative control | - |
| 22 | JA | 23.02447 | -81.95306 | 125 | NT-fl-xa | Crop | Thermoelectric power plant (A) | 15.1 |
| 23 | SJ | 22.94767 | -82.14096 | 150 | NT-fl-xa | Pasture | Cable industry (D) | 0.5 |
| 24 | SJ | 22.94931 | -82.14153 | 146 | NT-fl-xa | Pasture | Cable industry (D) | 0.5 |
| 25 | SJ | 22.94995 | -82.14541 | 140 | NT-fl-xa | Pasture | Cable industry (D) | 0.9 |
| 26 | SJ | 22.95512 | -82.14826 | 148 | NT-fl-xa | Pasture | Rubber manufactory (E) | 0.4 |
| 27 | SJ | 22.94067 | -82.09318 | 112 | NT-fl-ro | Pasture | Asphalt factory (C) | 1.3 |
| 28 | SJ | 22.93252 | -82.10400 | 123 | NT-fl-ro | Pasture | Asphalt factory (C) | 2.2 |
| 29 | SJ | 22.93351 | -82.08675 | 118 | NT-fl-ro | Crop | Asphalt factory (C) | 0.4 |
| 30 | SJ | 22.93561 | -82.08528 | 116 | NT-fl-ro | Forest | Asphalt factory (C) | 0.4 |
| 31 | SJ | 22.99024 | -82.21736 | 133 | NT-fl-ro | Pasture | Negative control | - |
| 32 | SJ | 22.94364 | -82.15568 | 158 | NT-fl-ro | Pasture | Waste incineration (F) | 0.3 |
| 33 | GU | 22.81318 | -82.03197 | 70 | NT-fl-ro | Crop | Traffic/roads | - |
| 34 | GU | 22.81380 | -82.03532 | 56 | NT-fl-ro | Pasture | Traffic/roads | - |
| 35 | GU | 22.74191 | -82.03600 | 29 | VR-pe-gl | Crop | Traffic/roads | - |
| 36 | GU | 22.86170 | -82.07363 | 87 | NT-fl-ro | Pasture | Traffic/roads | - |
| 37 | GU | 22.71817 | -82.03778 | 56 | GL-oy | Crop | Traffic/roads | - |
| 38 | GU | 22.87942 | -81.92469 | 115 | CM-dy | Pasture | Traffic/roads | - |
| 39 | SJ | 22.99199 | -82.15715 | 130 | NT-fl-ro | Pasture | Negative control | - |
| ^a^SC: Santa Cruz del Norte, JA: Jaruco, SJ: San José de las Lajas, GU: Güines. ^b^ LP-rz-ro: rhodic, rendzic leptosol, CM-dy: dystric cambisol, RG-sk: skeletic regosol, NT-fl-ro: rhodic, ferralic nitisol, NT-fl-xa: xanthic, ferralic nitisol, VR-pe-gl: gleyic, pellic vertisol, GL-oy: oxygleyic gleysol. Soil types are described in detail in Table S1. ^c^Land use: pasture = grazing land and crop = maize, sweet potato, and tomato. ^d^Letters behind the potential contamination and/or pollution source refer to letters in Figure 1 and 4. | | | | | | | | |

| **Table S4. Quality control and assurance parameters of the analytical methods used to quantify heavy metals in soils of Mayabeque, Cuba.** | | | | | | | | |
| --- | --- | --- | --- | --- | --- | --- | --- | --- |
| **Analyte** |  |  |  |  | **MR-863^d^** | | | |
|  | **MDL^a^ [mg/kg]** | **MQL^b^**  **[mg/kg]** | **Average blank [mg/kg]** | **Replicate numbers, (blank)** | **Average (Reference material) [mg/kg], Hg [µg/kg]^*^**  **n = 5** | **CV^c^ (Cuban Team) [%]**  **n = 5** | **Average (WEPAL)^e^ [mg/kg], Hg [µg/kg]** | **p-value^f^** |
| **Cd** | 0.08 | 0.1 | 0.17 | 21 | 0.5 | 6 | 0.8 | >0.99 |
| **Cr** | 0.08 | 0.17 | <0.08 |  | 39 | 3 | 40 | >0.99 |
| **Cu** | 0.3 | 0.7 | 0.39 |  | 28 | 2 | 32 | >0.99 |
| **Ni** | 0.04 | 0.12 | <0.04 |  | 30 | 2 | 33 | >0.99 |
| **Pb** | 0.55 | 0.93 | <0.55 |  | 68 | 1 | 70 | >0.99 |
| **Zn** | 1.94 | 2.71 | <1.94 |  | 149 | 1 | 162 | >0.99 |
| **Hg** | 0.002 | 0.003 | <0.09^a^ |  | 90 | 7 | 92 | >0.99 |
| ^a^ MDL: method detection limit, ^b^ MQL: method quantification limit, ^c^ CV: coefficient of variation, ^d^ MR-863: Control soil of inter-laboratory study, ^e^ WEPAL: Wageningen Evaluating programs for Analytical Laboratories (interlaboratory studies), ^f^ non-parametric ANOVA with the Kruskal Wallis method between the values of our laboratory* and WEPAL values | | | | | | | | |

| **Table S5. ANOVA model to test the influence of factors on heavy metal and iron concentrations. All concentrations were logarithmized to obtain evenly distributed residuals and qq-normal plots.** | |
| --- | --- |
| **Model** | **y_ijkl_ = µ + α_i_ + β_j_+ γ_k_ (αβγ)_ijk_ + ε_ijkl_** |
| **Anova** |  |
| **Variable** | **Explanation** |
| y | Dependent variable thus heavy metal concentrations |
| µ | Grand mean |
| α | Municipality |
| β | Soil type |
| γ | Land use (of soil) |
| αβγ | Interaction of municipality, soil type and land use incl. all combinations (αβ, αγ, γβ, αβγ) |
| ε | Residuals need to be normally distributed |
| i | 1,…, 4 (SC, JA, SJ, GU)^a^ |
| j | 1,…, 7 (LP-rz-ro, CM-dy, NT-fl-ro, NT-fl-xa, RG-sk, VR-pe-gl, GL-oy)^b^ |
| k | 1,…, 3 (pasture, crop, forest) |
| l | 1,…, n_ijk_ |

^a^ SC: Santa Cruz, JA: Jaruco, SJ: San José de las Lajas, GU: Güines. ^b^ LP-rz-ro: rhodic, rendzic leptosol, CM-dy: dystric cambisol, NT-fl-ro: rhodic, ferralic nitisol, NT-fl-xa: xanthic, ferralic nitisol, RG-sk: skeletic regosol, VR-pe-gl: gleyic, pellic vertisol, GL-oy: oxygleyic gleysol

Model and pairwise comparison outcomes according to Bonferroni of all heavy metals are listed below. If a factor (municipality, soil type, land use) was significant results of the t-test are indicated.

| **Influence of municipality, soil type, and land use on Cd** | | | |
| --- | --- | --- | --- |
|  | **p-value** | | |
| Municipality^a^ | 0.005721 ** | | |
| Soil type^b^ | 0.000235 *** | | |
| Land use | 0.179408 | | |
| Municipality*soil type | 0.418796 | | |
| Municipality* land use | 0.281451 | | |
| Soil type*land use | 0.840258 | | |
| Municipality*soil type*land use | 0.027243 * | | |
| Signif. codes: | <0.001***  <0.01**  ≤0.05* | | |
| ^a^municipality SC: Santa Cruz del Norte. JA: Jaruco. SJ: San José de las Lajas. GU: Güines | | | |
| **Pairwise comparisons of municipality using t-tests with pooled standard deviation (SD)** | | | |
| **p-value** | SC | JA | SJ |
| JA | 1.000 | - | - |
| SJ | 0.091 | 0.385 |  |
| GU | 1.000 | 1.000 | 1.000 |
|  | | | |
| **Pairwise comparisons of soil types using t-tests with pooled SD. RG-sk, VR-pe-gl, GL-oy soil types not considered** | | | |
| **p-value** | CM-dy | LP-rz-ro | NT-fl-ro |
| LP-rz-ro | 0.0070* | - | - |
| Nt-fl-ro | 0.0006** | 1.000 | - |
| Nt-fl-xa | 0.0023* | 1.000 | 1.000 |

| **Influence of municipality, soil type, and land use on Cr** | | | |
| --- | --- | --- | --- |
|  | **p-value** | | |
| Municipality^a^ | 0.21787 | | |
| Soil type^b^ | 0.00168 ** | | |
| Land use | 0.32826 | | |
| Municipality*soil type | 0.02968 * | | |
| Municipality* land use | 0.56042 | | |
| Soil type*land use | 0.39640 | | |
| Municipality*soil type*land use | 0.01595 * | | |
| Signif. codes: | <0.001***  <0.01**  ≤0.05* | | |
|  | | | |
| **Pairwise comparisons of soil types using t-tests with pooled SD. RG-sk, VR-pe-gl, GL-oy soil types not considered** | | | |
| **p-value** | CM-dy | LP-rz-ro | NT-fl-ro |
| LP-rz-ro | 0.0022* | - | - |
| Nt-fl-ro | 0.0024* | 1.000 | - |
| Nt-fl-xa | 0.0056* | 1.000 | 1.000 |

| **Influence of municipality, soil type, and land use on Cu** | | | | | | |
| --- | --- | --- | --- | --- | --- | --- |
|  | | **p-value** | | | | |
| Municipality^a^ | | 0.00149 ** | | | | |
| Soil type^b^ | | 0.01673 * | | | | |
| Land use | | 0.57114 | | | | |
| Municipality*soil type | | 0.21101 | | | | |
| Municipality* land use | | 0.71424 | | | | |
| Soil type*land use | | 0.98690 | | | | |
| Municipality*soil type*land use | | 0.63894 | | | | |
| Signif. codes: | | <0.001***  <0.01**  ≤0.05* | | | | |
|  | | | | | | |
| **Pairwise comparisons of municipality using t-tests with pooled SD** | | | | | | |
| **p-value** | | SC | | JA | | SJ |
| JA | | 1.000 | | - | | - |
| SJ | | 0.0175 | | 0.0064 | | - |
| GU | | 0.4983 | | 0.244 | | 1.000 |
| **Pairwise comparisons of soil types using t-tests with pooled SD. RG-sk, VR-pe-gl, GL-oy soil types not considered** | | | | | | |
| **p-value** | CM-dy | | LP-rz-ro | | NT-fl-ro | |
| LP-rz-ro | 0.6292 | | - | | - | |
| Nt-fl-ro | 1.0000 | | 0.0253* | | - | |
| Nt-fl-xa | 0.00095** | | 4.6e-0.6*** | | 0.0036* | |

| **Influence of municipality, soil type, and land use on Ni** | |
| --- | --- |
|  | **p-value** |
| Municipality | 0.0537 |
| Soil type | 0.0681 |
| Land use | 0.6859 |
| Municipality*soil type | 0.0535 |
| Municipality* land use | 0.4286 |
| Soil type*land use | 0.1538 |
| Municipality*soil type*land use | 0.0608 |

| **Influence of municipality, soil type, and land use on Pb** | | | |
| --- | --- | --- | --- |
|  | **p-value** | | |
| Municipality^a^ | 0.00694 ** | | |
| Soil type | 0.16347 | | |
| Land use | 0.19020 | | |
| Municipality*soil type | 0.26933 | | |
| Municipality* land use | 0.97205 | | |
| Soil type*land use | 0.66912 | | |
| Municipality*soil type*land use | 0.33969 | | |
| Signif. codes: | <0.001***  <0.01**  ≤0.05* | | |
|  | | | |
| **Pairwise comparisons of municipality using t-tests with pooled SD** | | | |
| **p-value** | SC | JA | SJ |
| JA | 0.3128 | - | - |
| SJ | 0.0042 | 0.7351 |  |
| GU | 0.1050 | 1.000 | 1.0000 |

| **Influence of municipality, soil type, and land use on Zn** | |
| --- | --- |
|  | **p-value** |
| Municipality | 0.333 |
| Soil type | 0.450 |
| Land use | 0.602 |
| Municipality*soil type | 0.114 |
| Municipality* land use | 0.897 |
| Soil type*land use | 0.334 |
| Municipality*soil type*land use | 0.704 |

| **Influence of municipality, soil type, and land use on Hg** | | | |
| --- | --- | --- | --- |
|  | **p-value** | | |
| Municipality^a^ | 0.000959 *** | | |
| Soil type | 0.747254 | | |
| Land use | 0.168576 | | |
| Municipality*soil type | 0.005381 ** | | |
| Municipality* land use | 0.153294 | | |
| Soil type*land use | 0.447833 | | |
| Municipality*soil type*land use | 0.830285 | | |
| Municipality^a^ |  | | |
| Signif. codes: | <0.001***  <0.01**  ≤0.05* | | |
|  | | | |
| **Pairwise comparisons of municipality using t-tests with pooled SD** | | | |
| **p-value** | SC | JA | SJ |
| JA | 1.000 | - | - |
| SJ | 0.045 | 0.014 | - |
| GU | 1.000 | 1.000 | 1.000 |

| **Influence of municipality, soil type, and land use on Fe** | | | |
| --- | --- | --- | --- |
|  | **p-value** | | |
| Municipality^a^ | 0.0239 * | | |
| Soil type^b^ | 0.3354 | | |
| Land use | 0.8639 | | |
| Municipality*soil type | 0.4214 | | |
| Municipality* land use | 0.1841 | | |
| Soil type*land use | 0.8676 | | |
| Municipality*soil type*land use | 0.8501 | | |
| Signif. codes: | <0.001***  <0.01**  ≤0.05* | | |
|  | | | |
| **Pairwise comparisons of municipality using t-tests with pooled SD** | | | |
| **p-value** | SC | JA | SJ |
| JA | 1.00 | - | - |
| SJ | 0.399 | 0.034 | - |
| GU | 0.510 | 0.071 | 1.000 |


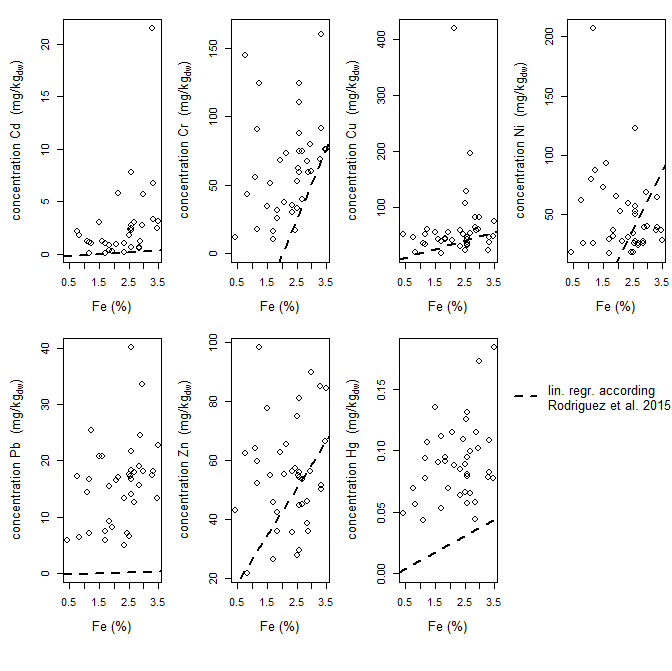


**Fig. S1:** Heavy metals opposed to soil iron (Fe) contents of Mayabeque, Cuba. Dashed lines indicate heavy metal and Fe contents according to linear regression models (Rodríguez et al., 2015). If data scatter above the dashed line an anthropogenic influence is indicated according Rodríguez et al. (2015).


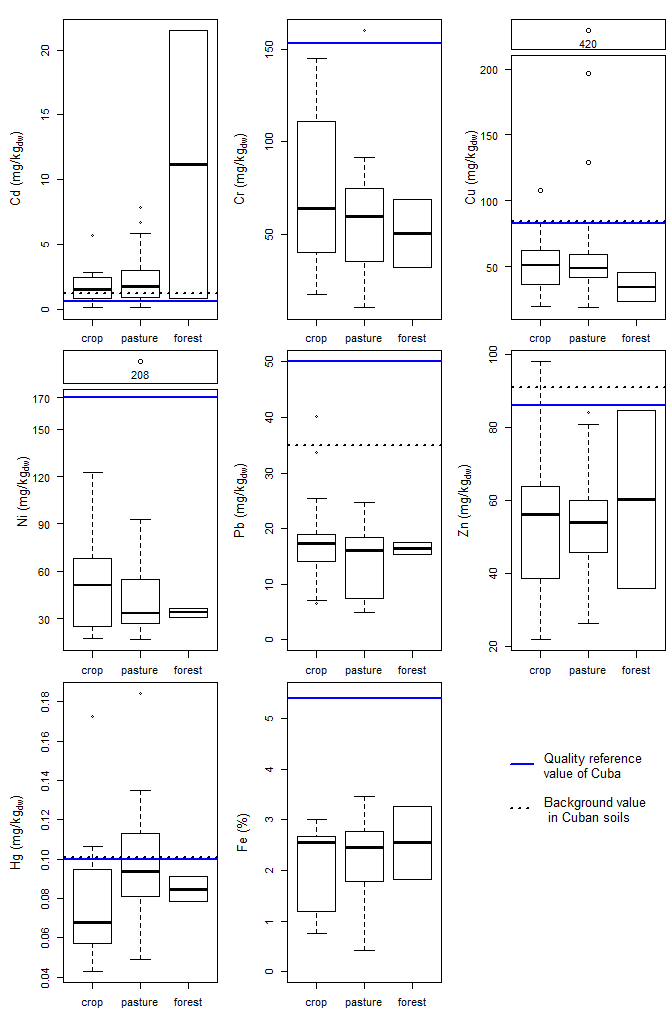


**Fig. S2:** Heavy metals in the soils of Mayabeque, Cuba, divided into different land uses (crop, n = 14, pasture, n= 23, forest, n = 2). The boxes represent the 25^th^ to 75^th^ percentile, the whiskers are the 10^th^ and 90^th^ percentile, and the dots are outliers. The bold black line in the box represents the median of the respective concentrations. The blue solid lines indicate quality reference value in Cuba (Rodríguez et al., 2015) and black dotted lines background values in Cuba (Rodríguez et al., 2015) (Table S7). The average background value of Cr is 463 mg/kg_dw_ and of Ni 294 mg/kg_dw_.


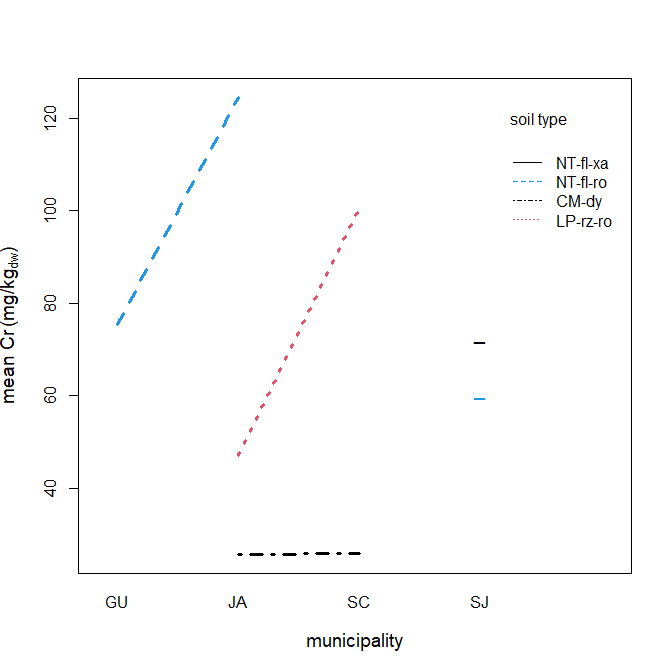


**Fig. S3:** Interaction plot of mean Cr concentrations in different municipalities that were divided according their soil types. Municipality abbreviations are Santa Cruz = SC, Jaruco = JA, San Jose = SJ, Guines = GU and soil types are xanthic, ferralic nitisols (NT-fl-xa, black line), rhodic, ferralic nitisols (NT-fl-ro, blue, dashed line), dystric cambisols (CM-dy, black, dotted, dashed line), and rhodic, rendzic leptosols (LP-rz-ro, red, dotted line). Due to their low number skeletic regosols (RG-sk), gleyic, pellic vertisol (VR-pe-gl), and oxygleyic gleysol were excluded from the analysis.


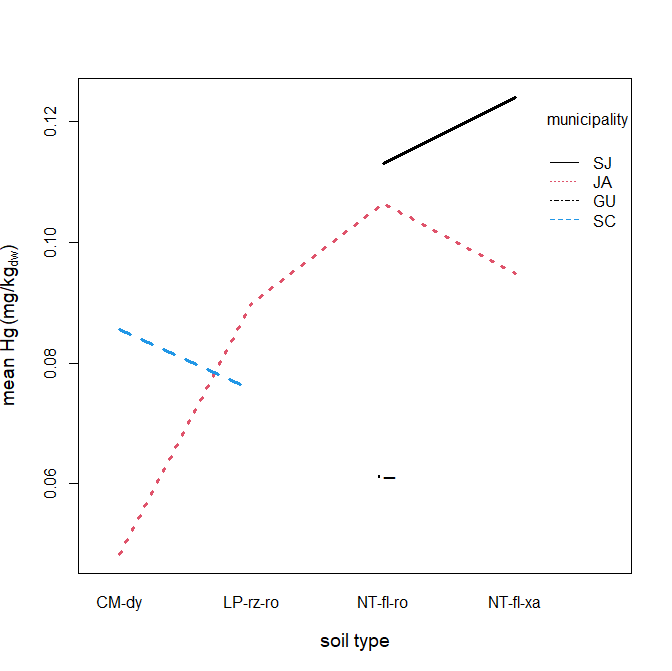


**Fig. S4:** Interaction plot of mean Hg concentrations in different soil types that were divided according their municipality. Soil type abbreviations are xanthic, ferralic nitisols (NT-fl-xa), rhodic, ferralic nitisols (NT-fl-ro), dystric cambisols (CM-dy), and rhodic, rendzic leptosols (LP-rz-ro) and municipalities are Santa Cruz = SC (black, solid line), Jaruco = JA (red, dotted line), Guines = GU (black, dashed, dotted line), San Jose = SJ (blue, dashed line). Due to their low number skeletic regosols (RG-sk), gleyic, pellic vertisol (VR-pe-gl), and oxygleyic gleysol were excluded from the analysis.

| **Table S6. Concentrations of heavy metals [mg/kg_dw_], texture [%], organic carbon (C_org_) [%], and Fe [%] in soils of Mayabeque, Cuba.** | | | | | | | | | | | | | | |
| --- | --- | --- | --- | --- | --- | --- | --- | --- | --- | --- | --- | --- | --- | --- |
| **Site number** | **Cd** | **Cr** | **Cu** | **Ni** | **Pb** | **Zn** | **Hg** | **pH** | **Soil type^a^** | **Sand %** | **Silt %** | **Clay %** | **C_org_ %** | **Fe %** |
| 1 | 3.34 | 160 | 38.5 | 64.3 | 18.1 | 51.7 | 0.11 | 7.92 | LP-rz-ro | 20.4 | 61.8 | 12.7 | 2.95 | 3.32 |
| 2 | 2.48 | 125 | 38.1 | 52.5 | 16.7 | 56.0 | 0.08 | 7.95 | LP-rz-ro | 17.3 | 65.6 | 10.2 | 4.00 | 2.57 |
| 3 | 1.76 | 52.6 | 24.5 | 33.4 | 6.62 | 27.9 | 0.09 | 7.89 | LP-rz-ro | 26.4 | 45.7 | 22.9 | 2.93 | 2.50 |
| 4 | 1.80 | 43.1 | 19.7 | 25.1 | 6.46 | 21.9 | 0.06 | 8.15 | LP-rz-ro | 36.9 | 35.4 | 23.4 | 2.51 | 0.84 |
| 5 | 2.23 | 145 | 47.9 | 61.2 | 17.2 | 62.3 | 0.07 | 8.01 | LP-rz-ro | 23.7 | 57.7 | 13.2 | 3.13 | 0.76 |
| 6 | 2.33 | 111 | 32.8 | 49.5 | 14.1 | 44.7 | 0.06 | 8.18 | LP-rz-ro | 21.8 | 62.2 | 10.6 | 3.11 | 2.59 |
| 7 | 0.42 | 25.4 | 43.1 | 36.0 | 9.20 | 42.3 | 0.09 | 7.37 | CM-dy | 13.8 | 28.9 | 52.0 | 3.06 | 1.85 |
| 8 | 0.09 | 17.3 | 53.2 | 25.4 | 7.12 | 59.6 | 0.08 | 7.43 | CM-dy | 15.5 | 13.4 | 66.7 | 2.53 | 1.18 |
| 9 | 0.30 | 67.9 | 56.4 | 65.3 | 8.12 | 62.8 | 0.07 | 7.22 | LP-rz-ro | 15.9 | 25.0 | 51.9 | 4.2 | 1.93 |
| 10 | 0.16 | 35.5 | 59.6 | 30.3 | 4.87 | 56.2 | 0.08 | 7.37 | CM-dy | 28.8 | 22.5 | 45.1 | 2.07 | 2.33 |
| 11 | ND^b^ | 16.6 | 52.0 | 17.7 | 7.02 | 57.3 | 0.11 | 6.65 | RG-sk | 23.6 | 19.3 | 53 | 2.38 | 2.46 |
| 12 | 1.00 | 30.4 | 31.0 | 59.0 | 13.4 | 35.6 | 0.06 | 7.91 | LP-rz-ro | 16.5 | 42.1 | 38.3 | 1.79 | 2.34 |
| 13 | 1.12 | 91.0 | 35.1 | 208 | 16.7 | 52.1 | 0.09 | 7.84 | LP-rz-ro | 18.7 | 38.2 | 38.6 | 2.61 | 1.17 |
| 14 | 1.25 | 51.3 | 43.3 | 93.0 | 20.8 | 55.1 | 0.09 | 7.30 | LP-rz-ro | 6.2 | 30.5 | 56.2 | 4.10 | 1.62 |
| 15 | 1.04 | 16.3 | 18.6 | 28.3 | 5.77 | 26.5 | 0.11 | 7.86 | LP-rz-ro | 17 | 39 | 41.8 | 1.30 | 1.71 |
| 16 | ND | 11.6 | 52.9 | 17.2 | 5.81 | 43.0 | 0.05 | 7.54 | CM-dy | 13.6 | 18.1 | 65.3 | 1.77 | 0.42 |
| 17 | 0.13 | 10.0 | 40.6 | 16.6 | 7.41 | 45.8 | 0.05 | 7.41 | CM-dy | n.d.^c^ | n.d. | n.d. | 2.13 | 1.71 |
| 18 | 1.19 | 55.5 | 36.2 | 79.3 | 14.4 | 63.9 | 0.04 | 8.02 | CM-dy | 10.7 | 41.6 | 44.8 | 1.66 | 1.12 |
| 19 | 1.07 | 125 | 61.3 | 86.7 | 25.5 | 98.1 | 0.11 | 6.79 | NT-fl-ro | 26.7 | 40.4 | 30.3 | 1.53 | 1.25 |
| 20 | 21.5 | 68.6 | 23.3 | 36.5 | 17.5 | 84.9 | 0.08 | 6.10 | RG-sk | 34.5 | 26 | 34.2 | 3.05 | 3.27 |
| 21 | 5.68 | 60.3 | 83.3 | 40.0 | 18.2 | 89.8 | 0.17 | 6.99 | NT-fl-ro | 32.9 | 46.2 | 17.5 | 1.97 | 3.00 |
| 22 | 0.63 | 88.0 | 43.9 | 122 | 40.2 | 29.6 | 0.09 | 7.25 | NT-fl-xa | 21.2 | 39.2 | 36.4 | 1.84 | 2.57 |
| 23 | 5.81 | 73.6 | 419.5 | 26.9 | 17.0 | 65.5 | 0.09 | 5.97 | NT-fl-xa | 24.1 | 15.2 | 56.8 | 2.27 | 2.16 |
| 24 | 3.02 | 74.3 | 196.5 | 24.8 | 12.6 | 53.7 | 0.10 | 5.67 | NT-fl-xa | 34.6 | 37.1 | 25.2 | 1.81 | 2.69 |
| 25 | 2.40 | 62.5 | 128.7 | 25.5 | 17.2 | 55.0 | 0.13 | 5.31 | NT-fl-xa | n.d. | n.d. | n.d. | 2.54 | 2.56 |
| 26 | 3.15 | 76.2 | 75.5 | 28.0 | 22.7 | 84.3 | 0.18 | 7.02 | NT-fl-xa | n.d. | n.d. | n.d. | 2.54 | 3.47 |
| 27 | 1.28 | 59.5 | 59.6 | 38.5 | 24.6 | 36.2 | 0.11 | 6.50 | NT-fl-ro | n.d. | n.d. | n.d. | 2.56 | 2.87 |
| 28 | 2.74 | 59.5 | 34.9 | 27.1 | 21.8 | 54.1 | 0.08 | 6.05 | NT-fl-ro | 36.4 | 28.9 | 32 | 1.59 | 2.57 |
| 29 | 2.80 | 80.0 | 62.1 | 68.1 | 33.7 | 56.4 | 0.10 | 7.14 | NT-fl-ro | 26.3 | 26.1 | 43.2 | 2.56 | 2.96 |
| 30 | 0.84 | 31.7 | 45.8 | 30.7 | 15.4 | 35.9 | 0.09 | 7.53 | NT-fl-ro | 13.9 | 40.4 | 40.6 | 2.93 | 1.83 |
| 31 | 7.81 | 75.0 | 48.1 | 56.4 | 18.4 | 81.0 | 0.13 | 6.29 | NT-fl-ro | 17.8 | 33.1 | 45.3 | 2.20 | 2.59 |
| 32 | 3.01 | 34.8 | 55.3 | 72.2 | 20.9 | 77.6 | 0.14 | 7.48 | NT-fl-ro | n.d. | n.d. | n.d. | 3.93 | 1.51 |
| 33 | 0.54 | 67.4 | 64.9 | 24.8 | 19.0 | 38.7 | 0.04 | 7.99 | NT-fl-ro | 24.8 | 53.7 | 19 | 1.47 | 2.85 |
| 34 | 0.68 | 67.5 | 83.2 | 27.3 | 15.6 | 46.0 | 0.06 | 8.01 | NT-fl-ro | 18.7 | 59.5 | 18.6 | 1.85 | 2.85 |
| 35 | ND | 39.8 | 54.2 | 23.6 | 18.0 | 45.0 | 0.06 | 7.88 | VR-pe-gl | 9.9 | 23.2 | 64.3 | 1.52 | 2.67 |
| 36 | 6.71 | 91.4 | 47.1 | 38.8 | ND | 50.3 | 0.08 | 7.86 | NT-fl-ro | 33.7 | 19.6 | 41.9 | 2.78 | 3.32 |
| 37 | ND | 33.2 | 108 | 17.2 | 17.5 | 75.0 | 0.07 | 7.86 | GL-oy | 12.1 | 19.6 | 66.2 | 1.23 | 2.52 |
| 38 | 0.91 | 37.3 | 42.3 | 52.5 | 16.5 | 55.1 | 0.12 | 6.79 | CM-dy | 17.5 | 24.5 | 53.2 | 2.78 | 2.09 |
| 39 | 2.46 | 75.8 | 48.5 | 36.2 | 13.3 | 66.3 | 0.08 | 6.68 | NT-fl-ro | n.d. | n.d. | n.d. | 2.20 | 3.44 |
| ^a^ soil type are explained and described in Table S2;  ^b^ ND: not detected; ^c^ n.d.: not determined | | | | | | | | | | | | | | |

| **Table S7. Heavy metal concentration ranges (min – max (mean)) in mg/kg dry weight (dw) in soil samples from Mayabeque, Cuba.** | | | | | | | | |
| --- | --- | --- | --- | --- | --- | --- | --- | --- |
| **Concentration (**mg/kg_dw_) | **Cd** | **Cr** | **Cu** | **Ni** | **Pb** | **Zn** | **Hg** | **Main potential**  **Contamination and/or pollution source(s)*** |
| **Data from this study** | | | | | | | | |
| SC (min - max (median) n=11) | 0.2 – 3.0 (1.8) | 16.6 - 161 (52.6) | 19.7 - 59.6 (43.1) | 17.7 - 65.3 (36) | 4.9 - 18.1 (8.1) | 21.9 - 62.8 (56) | 0.06 - 0.1 (0.08) | A |
| JA (min - max (median) n=10) | 0.1 – 21.6 (1.1) | 10 – 125 (53.4) | 18.6 - 61.3 (38.4) | 16.6 - 208 (69.1) | 5.8 - 40.2 (15.5) | 26.4 - 98.1 (48.9) | 0.04 - 0.1 (0.08) | A, B |
| SJ (min - max (median) n=12) | 0.8 - 7.8 (2.9) | 31.7 – 80.0 (68) | 34.9 - 420 (60.9) | 24.7 - 72.2 (33.4) | 12.6 - 33.7 (18.3) | 35.9 - 89.8 (60.9) | 0.1 - 0.2 (0.11) | C, D, E, F |
| GU (min - max (median) n=6) | 0.5 - 6.7 (0.8) | 33.2 - 91.4 (53.6) | 42.3 - 108 (59.6) | 17.2 - 52.5 (26) | 15.6 – 19.0 (17.5) | 38.7 – 75.0 (48.2) | 0.04 - 0.1 (0.07) | Traffic/roads |
| Control sites (background soils, min-max,) (n=4) | 0.09 - 5.7 | 17.3 - 75.8 | 48.5 - 83.2 | 25.4 - 65.3 | 7.1 - 18.2 | 59.6 - 89.8 | 0.07 - 0.2 |  |
| Range min – max ( median) over 39 sites | 0.1 - 21.6 (1.8) | 10.0 - 160 (60.3) | 18.6 - 420 (48.1) | 16.6 - 208 (36.2) | 4.9 - 40.2 (16.7) | 21.9 - 98.1 (55.0) | 0.04 - 0.2 (0.09) |  |
| **Data from the literature** | | | | | | | | |
| Quality Reference Value in Cuba (Rodríguez et al., 2015) | 0.6 | 153 | 83 | 170 | 50 | 86 | 0.1 |  |
| Average background values in Cuba (Rodríguez et al., 2015) | 1.2 | 463 | 84 | 294 | 35 | 91 | 0.1 |  |
| Crustal average concentrations (Kabata-Pendias, 2010) | 0.1 | 100 | 55 | 20 | 15 | 70 | 0.07 |  |
| Median concentrations for soils of Parana State, Brazil (Licht, 2005) | 0.2 | 86 | 109 | 25 | 22 | 73 | 0.05 |  |
| **Environmental Quality Standards** | | | | | | | | |
| Investigation value in Brazil (CONAMA, 2009) | 3 | 150 | 200 | 35 | 180 | 450 | 12 |  |
| Guide value in Switzerland consider uncontaminated soil (VBBo, 1998) | 0.8 | 50 | 40 | 50 | 50 | 150 | 0.5 |  |
| Clean-up values in Switzerland (VBBo, 1998) | l30 |  | 1000 |  | 2000 | 2000 |  |  |
| * A: thermoelectric power plant, B: zeolite production plant, C: asphalt factory, D: cable industry, E: rubber factory, F: waste incineration | | | | | | | | |

| **Table S8. Regression parameters of the relationship between heavy metals and Fe concentrations in soils of Mayabeque, Cuba.** | | | | | |
| --- | --- | --- | --- | --- | --- |
| Element | Slope coefficient | Constant | Standard error of estimate | R^2^ | p-value |
| Cd | 1.9E-04 | -1.72 | 7.9E-05 | 0.15 | p≤0.05 |
| Cr | 1.2E-03 | 36.42 | 7.6E-04 | 0.07 | p>0.05 |
| Cu | 8.0E-04 | 46.41 | 1.5E-03 | 0.009 | p>0.05 |
| Ni | -1.1E-03 | 71.95 | 7.6E-04 | 0.057 | p>0.05 |
| Pb | 3.3E-04 | 8.59 | 1.6E-04 | 0.12 | p≤0.05 |
| Zn | 3.3E-04 | 47.63 | 3.9E-04 | 0.02 | p>0.05 |
| Hg | 1.5E-06 | 0.06 | 6.2E-07 | 0.16 | p≤0.05 |

## Aim of a principal component analysis (PCA)

A PCA can only be fed with numerical inputs. The aim of a PCA is to reduce a multidimensional dataset comprising of variables (in this study all heavy metal concentrations, pH, distance to pollution source, organic carbon, texture, and Fe) and the observations (39 soil samples) but keep the variance as high as possible (Hilber et al., 2020). To obtain a maximum variance with a minimum of variables the ones with a short arrow need to be cancelled from the PC analysis. Additionally, two variables that correlate highly (r >0.9) only one should be used for the PCA as one can represent the other.

## S3. How to read a biplot

Vectors or arrows in the biplot as shown in Fig. 4 that are close together are heavy metal concentrations that correlate highly. Opposite vectors correlate negatively. Angles close to 90° show no or low correlation. Short arrows represent the heavy metal weakly in comparison to long arrows, which is the reason why the correlation coefficient between two short arrows is lower (e.g. Cd and Pb, r = 0.42, Table S10) than between two long arrows, although the angle between the latter two is bigger than the former two (e.g. Cr and Pb, r = 0.60, Table S10). Grey abbreviations in the biplot are observations. They indicate the municipality, the potential contamination and/or pollution source (A to F, Fig. 1) and the distance to it (Table S3). The upper x-axis and the right y-axis indicate the x/y scores of the vectors.

| **Table S9. Correlation coefficient matrix of logarithmized heavy metal and iron contents and soil properties such as pH, organic carbon (C_org_), texture (sand, silt, clay), and logarithmized distance to a potential pollution source in Mayabeque, Cuba. Where no pollution source was indicated (Table S3) an infinite value of 100 km was put. Correlation coefficients >0.5 are indicated in bold.** | | | | | | | | | | | | | | |
| --- | --- | --- | --- | --- | --- | --- | --- | --- | --- | --- | --- | --- | --- | --- |
|  | pH | C_org_ | Sand | Silt | Clay | Cd | Cr | Cu | Ni | Pb | Zn | Hg | Distance | Fe |
| pH | 1.00 |  |  |  |  |  |  |  |  |  |  |  |  |  |
| C_org_ | 0.15 | 1.00 |  |  |  |  |  |  |  |  |  |  |  |  |
| Sand | -0.39 | -0.34 | 1.00 |  |  |  |  |  |  |  |  |  |  |  |
| Silt | **0.59** | 0.03 | -0.05 | 1.00 |  |  |  |  |  |  |  |  |  |  |
| Clay | -0.34 | 0.05 | -0.43 | **-0.88** | 1.00 |  |  |  |  |  |  |  |  |  |
| Cd | -0.41 | 0.03 | 0.42 | 0.19 | -0.39 | 1.00 |  |  |  |  |  |  |  |  |
| Cr | -0.04 | 0.20 | 0.23 | **0.53** | **-0.61** | 0.49 | 1.00 |  |  |  |  |  |  |  |
| Cu | **-0.51** | -0.16 | 0.12 | -0.22 | 0.15 | 0.06 | 0.21 | 1.00 |  |  |  |  |  |  |
| Ni | 0.17 | 0.24 | -0.39 | 0.13 | 0.06 | 0.03 | 0.45 | -0.20 | 1.00 |  |  |  |  |  |
| Pb | -0.27 | -0.07 | 0.00 | 0.19 | -0.17 | 0.42 | **0.60** | 0.23 | 0.50 | 1.00 |  |  |  |  |
| Zn | **-0.52** | 0.10 | 0.06 | -0.18 | 0.12 | 0.30 | 0.38 | 0.41 | 0.22 | 0.35 | 1.00 |  |  |  |
| Hg | -0.47 | 0.01 | 0.10 | -0.23 | 0.16 | 0.27 | -0.02 | 0.15 | 0.14 | 0.16 | 0.30 | 1.00 |  |  |
| Distance | -0.12 | 0.05 | -0.12 | -0.33 | 0.35 | -0.08 | -0.17 | -0.19 | 0.32 | -0.16 | 0.29 | 0.30 | 1.00 |  |
| Fe | -0.32 | -0.06 | 0.20 | 0.13 | -0.21 | 0.32 | 0.16 | 0.20 | -0.20 | 0.27 | 0.09 | 0.26 | -0.23 | 1.00 |

| **Table S10. Identification of possible soil contamination and/or pollution sources according to Hämmann and Desaules (2003).** | | | | | | | |
| --- | --- | --- | --- | --- | --- | --- | --- |
| Soil containing pollutants | **Pb** | **Cd** | **Cr** | **Cu** | **Ni** | **Hg** | **Zn** |
| Surroundings of infrastructure |  | | | | | | |
| Roads | X | X |  |  |  |  | X |
| Waste Incineration | X | X |  | X |  | X | X |
| Electric power plant/station (furnaces excl. gas and extra-light heating oil)^a^ | X | X | X |  |  |  | X |
| Textile production |  |  | X | X |  |  |  |
| Soils of intensive cultivation | X |  |  | X |  |  |  |

^a^ the electric power plant burns oil to heat water to produce currency by steam.

**References**

CONAMA (2009). Conselho Nacional do Meio Ambiente, Resolução. Diário Oficial [da República Federativa do Brasil], Brasília 420, 81-84.

Hämmann, M., & Desaules, A. (2003). Sampling and sample pretreatment for soil pollutant monitoring. Published by the Swiss Agency for the Environment, Forests and Landscape SAEFL, 2003 Berne, Switzerland soil sampling manual OIS, 104.

Hernández-Jiménez, A., Pérez Jiménez, J.M., Bosch Infante, D., & Castro Speck, N., (2015). Clasificación de los suelos de Cuba 2015. Instituto Nacional de Ciencias Agrícolas, Instituto de Suelos, Ediciones INCA, Cuba, p. 93.

Hilber, I., & Gabbert, S. (2020). Choosing the best for preventing the worst: A structured analysis of the selection of risk management options in REACH restriction dossiers. Regulatory Toxicology and Pharmacology 118, 104809.

IUSS Working Group WRB, (2015). World reference base for soil resources 2014 International soil classification system for naming soils and creating legends for soil maps. Update 2015.

Kabata-Pendias, A., (2010). Trace elements in soils and plants.

Licht, O. (2005). Geoquímica de solos do Estado do Paraná, vol 1/2. Mineropar, Curitiba, Paraná.

Lopez-Kramer, J., Gutierrez Betty, Jaimez Efrén, Guerra Mario, Rocamora Ernesto, Sainz Yolanda, Díaz Idelfonso, Acosta Fausto, Alcaide José, Valdés MaráG, & Polo Bárbara (2012). Caracterización geoambiental del municipio Santa Cruz del Norte, Provincia Mayabeque, Cuba. Ciencias de la Tierra y el Espacio 13, 25-35.

Rodríguez, M., Montero, A., Muñiz, O., Araújo, C., Calero, B., Aguiar, A., Miranda, C., & Agra, Y. (2015). Background concentrations and reference values for heavy metals in soils of Cuba. Environmental Monitoring and Assessment 187, 1-10.

VBBo (1998). Verordnung über Belastungen des Bodens. 814.12, 1-12.
